# Drivers of genomic loss of heterozygosity in leiomyosarcoma are distinct from carcinomas

**DOI:** 10.1038/s41698-022-00271-x

**Published:** 2022-04-25

**Authors:** Nathan D. Seligson, Joy Tang, Dexter X. Jin, Monica P. Bennett, Julia A. Elvin, Kiley Graim, John L. Hays, Sherri Z. Millis, Wayne O. Miles, James L. Chen

**Affiliations:** 1 Department of Pharmacotherapy and Translational Research, The University of Florida, Jacksonville, FL USA; 2 Department of Pharmacogenomics and Translational Research, Nemours Children’s Specialty Care, Jacksonville, FL USA; 3 Division of Medical Oncology, Department of Internal Medicine, The Ohio State University, Columbus, OH USA; 4 Foundation Medicine Inc, Cambridge, MA USA; 5 Department of Computer and Information Science and Engineering, The University of Florida, Gainesville, FL USA; 6 Division of Gynecologic Oncology, Department of Obstetrics and Gynecology, The Ohio State University, Columbus, OH USA; 7 Department of Cancer Biology and Genetics, The Ohio State University, Columbus, OH USA; 8 Department of Biomedical Informatics, The Ohio State University, Columbus, OH USA

**Keywords:** Cancer genomics, Sarcoma, Sarcoma

## Abstract

Leiomyosarcoma (LMS) is a rare, aggressive, mesenchymal tumor. Subsets of LMS have been identified to harbor genomic alterations associated with homologous recombination deficiency (HRD); particularly alterations in *BRCA2*. Whereas genomic loss of heterozygosity (gLOH) has been used as a surrogate marker of HRD in other solid tumors, the prognostic or clinical value of gLOH in LMS (gLOH-LMS) remains poorly defined. We explore the genomic drivers associated with gLOH-LMS and their clinical import. Although the distribution of gLOH-LMS scores are similar to that of carcinomas, outside of *BRCA2*, there was no overlap with previously published gLOH-associated genes from studies in carcinomas. We note that early stage tumors with elevated gLOH demonstrated a longer disease-free interval following resection in LMS patients. Taken together, and despite similarities to carcinomas in gLOH distribution and clinical import, gLOH-LMS are driven by different genomic signals. Additional studies will be required to isolate and confirm the unique differences in biological factors driving these differences.

## Introduction

Soft-tissue sarcomas (STS) comprise a group of rare mesenchymal tumors that constitute approximately 1% of all adult cancers worldwide^1^. Leiomyosarcomas (LMSs) are one of the most common subtypes of STS, comprising nearly 25% of STS. Although surgery is considered the standard of care therapy, LMS tumors recur in over 80% of persons with LMS even after adequate resection. Persons with unresectable or recurrent LMS have limited systemic therapeutic options and display widely varying responses to therapy^1–3^. To date, there are no approved biomarkers of response in LMS that can be used to guide clinical treatment. Furthermore, LMS demonstrates fewer genomic alterations than many other tumor types^4^. Although molecular subtypes of LMS have been proposed previously, their clinical relevance with regard to targeted therapy remains to be borne out^5–7^. Taken together, there is an urgent clinical need to identify prognostic and predictive biomarkers to guide the treatment of LMS.

Previous analysis has identified homologous recombination DNA repair (HR) pathway deficiencies (HRD) in LMS^8–13^. HRD describes a cellular phenotype that results in high levels of genomic scarring and instability^14^ and can be inferred by genomic alterations of genes vital to HR pathway efficiency or by genomic loss of heterozygosity (gLOH), telomeric allelic imbalance, large-scale state transitions, or a combination of these genomic measurements^15^. The best-described etiology of HRD is through alterations in genes involved in the HR pathway. A number of clinical trials have tested the utility of high gLOH or HRD scores to predict tumor response in non-LMS tumors^16–19^. The ARIEL2 ovarian cancer trial evaluated the use of the poly-(ADP-ribose) polymerase (PARP) inhibitor, rucaparib, in previously treated patients and found that patients with wild-type *BRCA1* or *BRCA2* (*BRCA1/2*) genes and high levels of gLOH were more likely to respond to PARP inhibition and had longer progression-free survival compared to patients with non-elevated gLOH levels^19,20^. Additional mutations associated with HRD have also been described, including *ATM*, *ATR*, *BRIP1*, *CHEK2*, and *NBN*, as well as the *RAD51* and *FANC* families of genes. In the REAL3 trial, patients with oesophagogastric cancer that had high levels of gLOH in their tumors showed improved treatment responses to platinum-based chemotherapy relative to patients with lower levels of gLOH^21^.

Genomic alterations in either *BRCA1* or *BRCA2* have been highly associated with HRD in many cancer types^22^. Although *BRCA1/2* alterations are unevenly shared among cancer types^23,24^, alterations in these genes are often predictive of response to PARP inhibitors in *BRCA1* or *BRCA2*-associated malignancies^25–28^. Previous studies of LMS have identified that more than a quarter (26%) of LMS tumors harbored a genomic alteration in the HR pathway, including homozygous deletion of *BRCA2* (3%)^9^. Uterine LMS (uLMS), demonstrates a significant enrichment of tumors exhibiting homozygous deletion of *BRCA2* (10%) and has been linked to sensitivity to PARP inhibition^9,10,29^.

Although, *BRCA2* loss and HRD have been identified in LMS, the clinical meaning of HRD in LMS and its association with gLOH are poorly understood. In this study, we present a large-scale genomic analysis of gLOH and the HR pathway in LMS. We note that genes associated with gLOH in LMS differ substantially from that of published data in carcinomas and provide evidence that gLOH may be associated with clinical prognosis in LMS.

## Results

### Patient and tumor characteristics

Genomic profiling data from 2478 individual LMS tumors were collected with 1658 meeting the criteria for calculation of gLOH (Supplementary Data 1). Tumors included represented a single tumor for each patient with no repeat testing included in this dataset. The most common primary site of these tumors was uLMS (*n* = 651, 39.3%); the remaining samples were classified as non-uterine LMS (non-uLMS). The most common sites of biopsy were retroperitoneum (*n* = 565), non-specified soft tissue (*n* = 301), lung (*n* = 190), and liver (*n* = 160). The majority of subjects in this cohort were female (*n* = 1331, 80.3%) with males representing 19.7%. Subjects represent diverse ages, ranging from 19 to 85 years at time of sequencing. Full demographic characteristics are available in Table 1.

**Table 1:** Subject demographics (*n* = 1658).

*Sex*	
Female	1331 (80.3%)
Male	327 (19.7%)
Age at sequencing (mean [SD])	58 [11.9] years
Uterine Disease	651 (39.3%)
*MSI status*	
High	7 (0.4%)
Stable	1643 (99.1%)
Not Performed	8 (0.5%)
*Tumor mutation burden (TMB)*	
TMB (mean [SD])	2.4 [4.0] Mutations/megabase
TMB ≥ 10 Mutations/Megabase	34 (2.1%)
gLOH (mean [SD])	12.9 [6.9]%

*MSI* microsatellite Instability, *gLOH* genomic loss of heterozygosity.

### gLOH distribution and percentage of gLOH high tumors

**Fig. 1:**
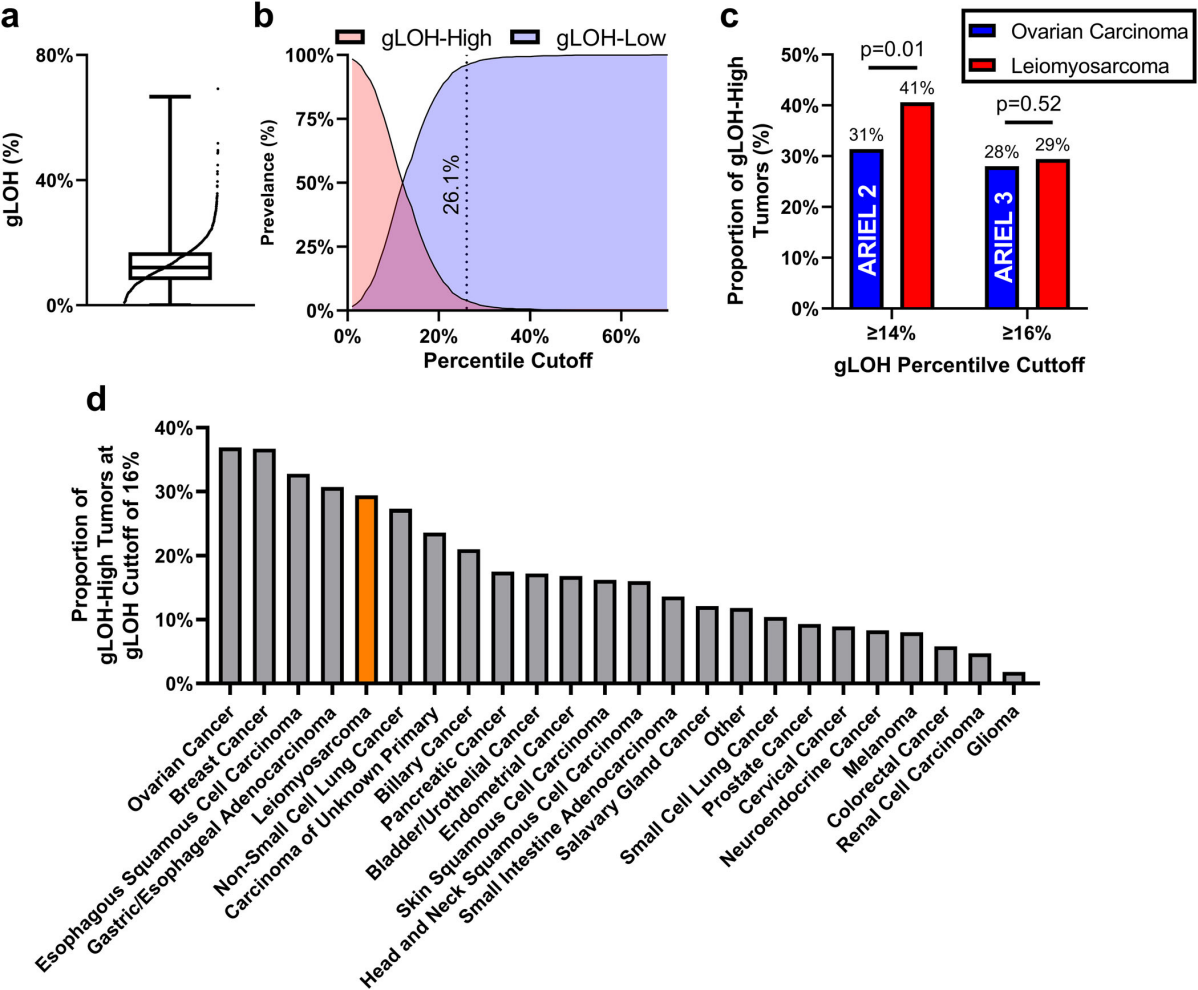
Distribution of gLOH in LMS. **a** LMS tumors display a mean gLOH of 12.9% and a log-normal distribution. Boxplot elements: center line—median, bounds of box—inter quartile rage, whiskers—minimum and maximum. **b** Prevalence of gLOH-High and gLOH-low tumors across the dataset identified an inflection point at 26.1%. **c** Compared to ovarian carcinoma from the ARIEL2 and ARIEL3 trials^20,25^, LMS demonstrated a higher or equal proportion of gLOH-High tumors at a gLOH cutoff of 14% and 16%, respectively. **d** Across a variety of cancer types, at a gLOH cutoff of 16%, LMS tumors from this analysis demonstrated similar gLOH-High distributions to cancers associated with significant proportions of HRD tumors previously reported^22^.

Our cohort of LMS tumors display a mean gLOH of 12.9% and a log-normal distribution with an inflection point at 26.1% (Fig. 1a). Using the inflection point as the marker of high LOH, only a small subset of 65 (3.9%) tumors met this gLOH cutoff (Fig. 1b). Data from the ARIEL2 and ARIEL3 PARP inhibitor clinical trial in patients with ovarian carcinoma proposed a gLOH cutoff of 14% and 16%, respectively^20,25^. Compared to ovarian carcinoma from these two trials, LMS demonstrated higher or equal proportion of gLOH-high tumors (Fig. 1c). Importantly, we note that this LMS cohort had comparable gLOH-High distributions to carcinomas associated with therapeutically targetable HRD phenotypes (Fig. 1d)^22^.

### Demographic correlation with gLOH

We next determined whether patient demographics contributed to gLOH scores in LMS. From this analysis, we found that patient sex was not associated with gLOH (Supplementary Fig. 1a); however, uLMS was correlated with elevated gLOH levels (Supplementary Fig. 1b) independent of sex (Supplementary Fig. 1c). Analysis of additional factors including age (Supplementary Fig. 1d), tumor mutational burden (Supplementary Fig. 1e), or microsatellite status (Supplementary Fig. 1f), revealed that these parameters do not correlate with gLOH. Given the elevated gLOH levels in uLMS tumors, we compared the genomic landscape of uLMS and non-uLMS tumors to identify differential prevalence of genomic alterations by tumor origin (Supplementary Table 1, Supplementary Fig. 1g). uLMS exhibited an increased prevalence of *ATRX* mutations (*q* = 2.4 × 10^−7^) and *RB1* homozygous deletion (*q* = 2.7 × 10^−6^). In contrast, non-uLMS tumors demonstrated an increased prevalence of *RB1* mutations (*q* = 1.3 × 10^−8^).

### Only BRCA2 homozygous deletion is highly associated with elevated gLOH

**Fig. 2:**
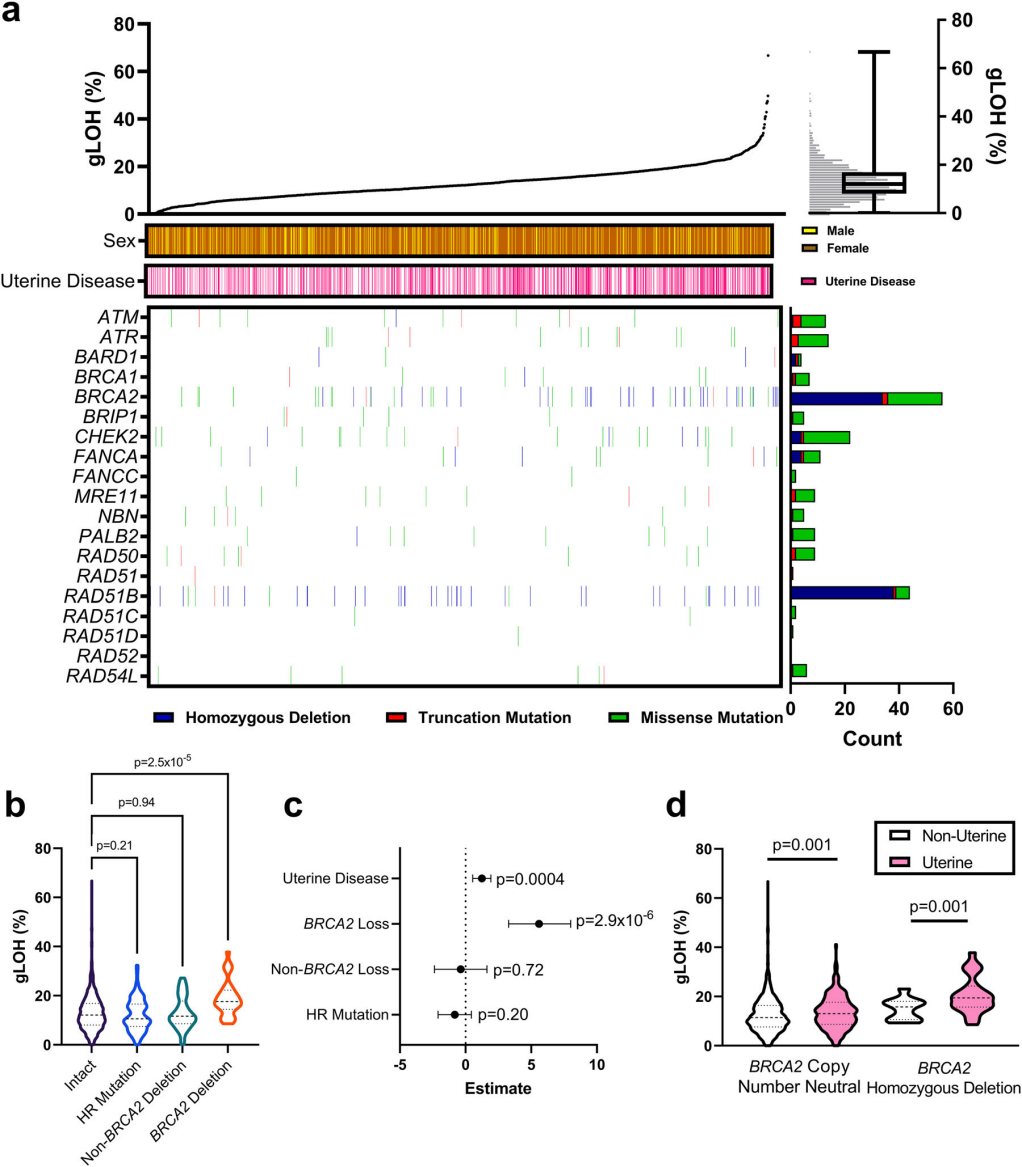
Contribution of the homologous recombination pathway on genomic loss of heterozygosity. **a** Genomic variants in the homologous recombination (HR) pathway were present across 1658 leiomyosarcomas (LMS). Tumors were sorted from lowest to highest gLOH. Boxplot elements: center line—median, bounds of box—inter quartile rage, whiskers—minimum and maximum. **b** Genomic alterations in the HR pathway were divided into three groups: HR mutations that included mutations in any gene of the HR pathway (*n* = 125), non-*BRCA2* homozygous deletion (*n* = 48), and *BRCA2* homozygous deletion (*n* = 34) (ANOVA, *p* = 5.5 × 10^−6^). Only *BRCA2* homozygous deletion was associated with a significant increase in LOH compared to tumors without any genomic alterations in the HR pathway (Mean ± SD, Welch’s *t* test; HR Intact *n* = 1451, 12.9 ± 6.9%; HR Pathway Mutation *n* = 48, 12.1 ± 6.5%, *p* = 0.21; Non-*BRCA2* homozygous deletion *n* = 48, 12.9 ± 6.6%, *p* = 0.94; *BRCA2* homozygous deletion *n* = 34, 18.9 ± 7.2%, *p* = 2.5 × 10^−5^). **c** Multivariate logistic regression identifies homozygous copy number loss of *BRCA2* (Estimate 5.6, 95% Confidence Interval [95%CI] 3.3–8.0, *p* = 2.9 × 10^−6^) and uterine disease status (Estimate 1.2, 95%CI 0.6–1.9, *p* = 0.0004) as independently associated with an increased LOH. **d** In LMS with intact *BRCA2* or homozygous deletion of *BRCA2*, uterine disease was independently associated with an increased LOH (Mean ± SD; *BRCA2* intact non-uLMS *n* = 997, 12.4 ± 7.0%; *BRCA2* intact uLMS *n* = 627, 13.5 ± 6.7%, Welch’s *t* test *p* = 0.001; homozygous copy number loss of *BRCA2* non-uLMS *n* = 10, 14.9 ± 4.5%; homozygous copy number loss of *BRCA2* uLMS *n* = 24, 20.6 ± 7.5%, Welch’s *t* test *p* = 0.03).

Across cancer types, genomic alterations in the HR pathway have been associated with an increased gLOH. We created a list of HR pathway genes based on prior published reports (“Methods”, Supplementary Table 2), from which we identified genetic changes in 12.5% of LMS tumors (Fig. 2a). The most prevalent genomic alterations identified were homozygous deletion of *RAD51B* (2.3%) and *BRCA2* (2.1%) (Fig. 2a); however, only homozygous deletion of *BRCA2* was correlated with elevated gLOH in LMS (Supplementary Fig. 2).

Univariate linear regression was then used to correlate gLOH levels with genomic alterations (Amplification, Homozygous Deletion, Rearrangement Mutations, Truncation Mutations, and Missense Mutations) for each gene in the HR pathway (Supplementary Table 3). In this analysis, only homozygous deletion of *BRCA2* retained statistical significance after correction for multiple comparisons. As HR pathway genomic alterations are infrequent in this dataset and individually do not correlate with gLOH, we combined tumors with non-*BRCA2* homozygous deletions together for further analysis. This was done to determine whether HR pathway changes in general, rather than specific alterations, were correlated to gLOH. When comparing mutations in the HR pathway, non-*BRCA2* homozygous deletion, and *BRCA2* homozygous deletion, only *BRCA2* homozygous deletion was associated with an increased gLOH (Fig. 2b). In a multivariate linear regression model, both *BRCA2* homozygous deletion and uterine disease status were independently associated with increased gLOH (Fig. 2c). Consistent with our previous reporting^9^, *BRCA2* homozygous deletion was more prevalent in uLMS (3.7%) than non-uLMS (0.9%). Interestingly, uLMS was associated with an increased gLOH in both *BRCA2* copy number neutral and *BRCA2* homozygous deletion tumors (Fig. 2d).

### Development of a LMS-specific gLOH signature

**Fig. 3:**
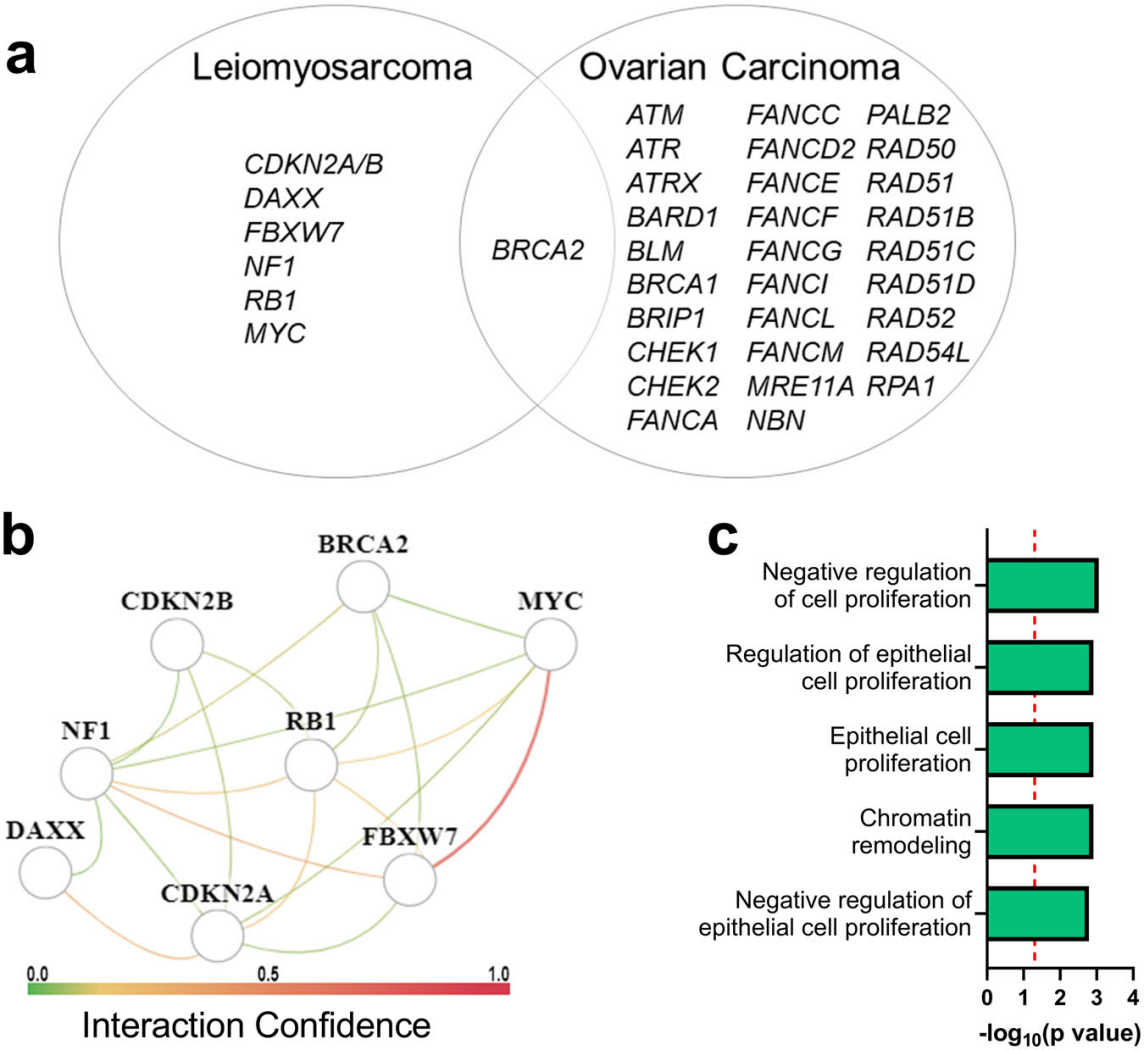
Genomic Associations with gLOH is Unique in LMS. **a** In ovarian carcinoma, gLOH has been associated with a number of genes associated with the HR and other DNA repair pathways. Alterations in these genes have further been associated with response to PARP inhibition. Gene alterations correlated to gLOH in LMS were notably different from those identified in ovarian carcinoma^25^. **b**, **c** Tissue-specific network analysis using humanbase identified common signaling connections between twelve genomic alterations associate with increased gLOH. Biological process enrichment identified patterns of cell proliferation and chromatin remodeling in this gene set.

To identify a genomic signature of gLOH in LMS, we first identified all candidate genes that met a threshold of a *q* < 0.1 and a prevalence of ≥0.5% with gLOH (Supplementary Table 4). Next, we fit a multivariate linear regression model including uterine disease status (Table 2) which identified eight genomic alterations that associated with increased gLOH, independent of uterine disease status, including homozygous deletion of *BRCA2*, *CDKN2A*, *CDKN2B, DAXX*, *NF1*, and *RB1*, as well as amplification of *FBXW7*, and *MYC*. In ovarian carcinoma, gLOH has been associated with a number of genes associated with the HR and other DNA repair pathways^25^. Alterations in these genes have further been associated with response to PARP inhibition. It is notable that of the genes associated with gLOH and PARP response in ovarian carcinoma, only *BRCA2* was associated with gLOH in this LMS cohort (Fig. 3a).

**Table 2:** Pathway agnostic linear regression of genomic loss of heterozygosity.

Gene	Alteration	Prevalence	Univariate linear regression			Multivariate linear regression	
			Estimate (95%CI)	*p*-value	Adjusted *p*-value	Estimate (95%CI)	*p*-value
*FBXW7*	Amplification	0.5%	18.75 (13.99–23.52)	2.0 × 10^−14^	1.5 × 10^−11^	16.1 (11.46–20.74)	1.4 × 10^−11^
*NF1*	Deletion	1.2%	10.67 (7.65–13.69)	6.3 × 10^−12^	4.5 × 10^−09^	9.63 (6.71–12.56)	1.3 × 10^−10^
*BRCA2*	Deletion	2.1%	6.13 (3.77–8.48)	3.7 × 10^−7^	2.7 × 10^−4^	9.63 (6.71–12.56)	2.3 × 10^−5^
*RB1*	Deletion	31.1%	1.9 (1.11–2.69)	2.3 × 10^−6^	1.7 × 10^−3^	1.62 (0.92–2.33)	6.4 × 10^−6^
*CDKN2A*	Deletion	7.1%	3.13 (1.82–4.43)	2.7 × 10^−6^	1.9 × 10^−3^	2.82 (1.57–4.08)	1.1 × 10^−5^
*CDKN2B*	Deletion	5.9%	3.11 (1.7–4.53)	1.6 × 10^−5^	0.002	–	
*MYC*	Amplification	1.8%	5.44 (2.93–7.95)	2.3 × 10^−5^	0.02	3.85 (1.45–6.26)	1.7 × 10^−3^
*DAXX*	Deletion	0.5%	9.54 (4.99–14.08)	4.0 × 10^−5^	0.03	8.34 (4.01–12.67)	1.6 × 10^−4^
Uterine LMS						0.86 (0.2–1.53)	0.01

To identify the network connections between genes correlated in the multivariate analysis, we used HumanBase tissue-specific network analysis (Fig. 3b). The direct interaction with the greatest confidence was between *MYC* and *FBXW7* (0.73). Previous reports suggest that elevated expression of *FBXW7* stabilizes *MYC* and may work to drive cell cycle progression^30^. All other interactions demonstrated a confidence between 0.33 and 0.05 and are noted in (Supplementary Table 5) Biological process enrichment identified patterns of cell proliferation and chromatin remodeling as most significantly enriched in this gene set (Fig. 3c). Mutual exclusivity was identified between homozygous deletion of *CDKN2A*/*B* and *RB1* (Supplementary Fig. 3a). Homozygous deletion of *BRCA2* was correlated with an increased prevalence of *RB1* homozygous deletion (Supplementary Fig. 3b). Despite this overlap, homozygous deletion of *BRCA2* and *RB1* were both independently linked to elevated gLOH (Supplementary Fig. 3c). In all evaluable instances, homozygous deletion of *BRCA2* and *RB1* were independent events. Even with close genomic proximity of *BRCA2* (13q13.1) and *RB1* (13q14.2), we find no evidence of large segment deletion driving the correlation between homozygous deletion of these genes in this dataset. Genomic alterations associated in this analysis with elevated gLOH together accounted for a statistically significant increase in gLOH (Supplementary Fig. 3d).

## Discussion

In different tumor types, gLOH has been used as a phenotypic biomarker of HRD. The cutoff at which gLOH represents HRD is unique to each cancer type and varies widely^21,25,31^. Accurately determining this point and its clinical utility in each tumor type is essential for measuring prognostic or biomarker potential. In this study, we demonstrate that LMS shares similarities to carcinomatous tumors in the distribution of gLOH. In our LMS cohort, the mean gLOH was 12.9%. Based on this analysis, the distribution of gLOH in LMS was similar to ovarian carcinoma, with ~30% of tumors identified to be gLOH-High using a cutoff of 16%^25^. We also found that uLMS had an elevated gLOH compared to non-uLMS, supporting previous data suggesting that uLMS is both clinically and molecularly distinct from non-uLMS^32^. Our data suggest that the genes contributing to the HR pathway may be differentially dysregulated across uterine-specific subtypes of LMS. This appears to be concordant with other tumor types.

Also concordant is that genomic variants in the HR pathway genes in LMS are prominently driven by variants in *BRCA1* and *BRCA2*. To date, over ten individual patient case studies have been published demonstrating clinical responses to PARP inhibitors in LMS tumors harboring homozygous deletions of *BRCA2*^9,10,29^. These promising observations necessitate identifying other genomic alterations present in a large patient cohort of LMS that may indicate sensitivity to PARP inhibition. In this study, we identified a prevalence of pathogenic HR gene alterations in <10% of all LMS. Homozygous deletion of *BRCA2* (*n* = 34) or *RAD51B* (*n* = 38) were the only highly recurrent HR gene alterations identified. Furthermore, only homozygous deletion of *BRCA2* was associated with an increase in gLOH. While further research is necessary to identify targetable genomic alterations in the HR pathway in LMS, although it is clear that a clinical focus should be put on patients whose tumors harbor homozygous deletion of *BRCA2*.

**Fig. 4:**
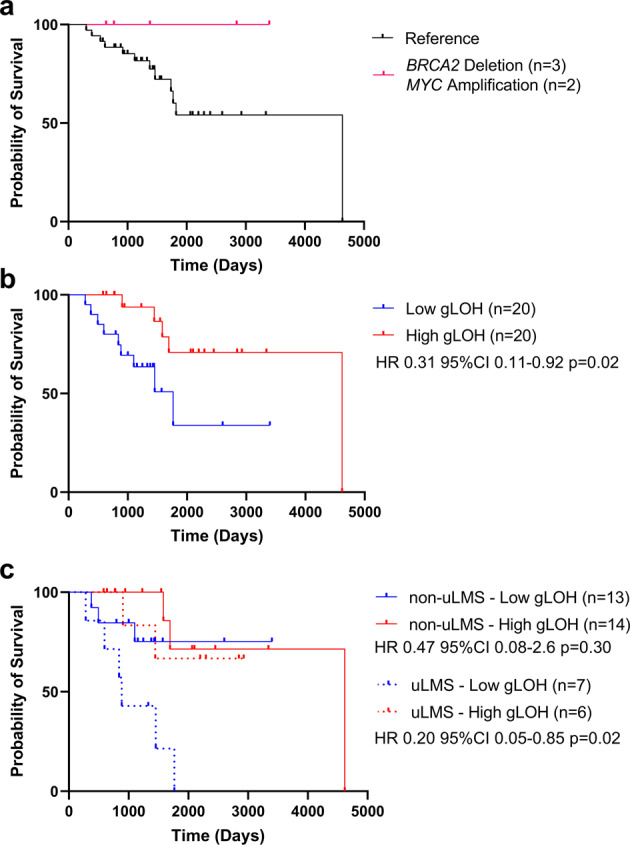
Elevated gLOH is a Positive Prognostic Indicator in Non-metastatic LMS. To assess the prognostic value of gLOH in LMS, we analyzed a sub-cohort of 40 patients with early stage LMS treated at the Ohio State University Comprehensive Cancer Center, whose tumors were sequenced by Foundation Medicine. In total, 40T > 1 LMS non-metastatic at diagnosis were collected. All subjects were treated initially with total resections. **a** While too few genomic alterations were present in the other LOH in LMS gene signature genes to conduct survival analysis, subjects whose tumors harbored homozygous deletion of *BRCA2* or amplification of *MYC* survived through the end of data collection. **b** gLOH scores above the group median (9.9%) were associated with an improved prognosis (HR 0.31, 95%CI 0.11–0.94, *p* = 0.02). **c** In uLMS, elevated gLOH was highly correlated with improved survival (HR 0.20 95%CI 0.05–0.85, *p* = 0.02). This effect was not identified in non-uLMS (HR 0.47 95%CI 0.08–2.6 *p* = 0.30).

To preliminarily assess the clinical consequences of elevated gLOH or genomic alterations associated with elevated gLOH in LMS, we performed a limited evaluation of an internal cohort of 40 early stage LMS tumors (IRB-2014C0181). Here we noted that elevated gLOH scores were associated with an improved prognosis (Fig. 4, Supplementary Table 6). As has also been previously reported in carcinomas, the clinical impact of the HR pathway may differ depending on tumor stage and/or clinical treatment course^21,33,34^. Prospective analysis of the role of the HR pathway in clinical outcomes for both early and late-stage LMS is warranted.

When we assessed the correlation between all pathogenic genomic alterations present in this dataset and gLOH, we identified correlations with elevated gLOH and genomic alterations in *RB1*, *CDKN2A/B, MYC, FBXW7*, and *NF1*. These genes differ from those previously identified in other tumors. Previous reports suggest that elevated expression of *FBXW7* stabilizes *MYC* and may work to drive cell cycle progression^30,35^. These genes share activity in the regulation of proliferation signaling and cell cycle progression. Unsurprisingly, tumors containing the homozygous deletion of *CDKN2B* also had deletion of *CDKN2A*. In LMS, disruption of cell cycle fidelity has been linked to HRD status, and likely allows cell cycle progression despite DNA damage and genome instability. It is possible that in LMS tumors with high accumulation of DNA damage, disruption of cell cycle checkpoint regulation through *RB1*, *CDKN2A/B, MYC, FBXW7*, or *NF1* is necessary to maintain the viability of the tumor. Further analysis of the interaction between DNA damage repair and cell cycle regulation in LMS is necessary. Additionally, alterations in *DAXX*, a gene required for genome stability, have previously been associated with PARP sensitivity^36^, were correlated with elevated gLOH in our LMS tumors. A notable absence from this list was *TP53*. While altered in 72.9% of tumors, no alteration in *TP53* was associated with gLOH scores. Previous studies in LMS and other cancers have correlated *TP53* alterations with gLOH or other measures of DNA damage^11,23,37^. The lack of association may be related to LMS-specific biology or differences in methodology of calculating gLOH/HRD. Identification of a mechanism linking *TP53* alterations and gLOH is a key area of study for future interpretation of these results. Further research to determine where gLOH may provide therapeutic opportunities, particularly with PARP inhibitors, to patients with this rare malignancy.

Our data interpretation has its limitations. First, the genomic analysis conducted included targeted next-generation DNA sequencing at high depth without a matched normal sample. Potentially key information that would be provided through genes not included in this targeted panel as well as somatic status of the variants identified was not available for analysis in this study. This impacts both the calculation of gLOH, which in this study is limited to a selection of >3,500 genomic sites, as well as which gene alterations can be associated with gLOH elevation. While it is impossible to know the impact that this limitation has on the validity of this study, further analysis should include at minimum a matched normal staple for somatic status calling and should consider broader sequencing to include either a whole genome or whole exome approach. Second, the clinical data available for this study were collected retrospectively from data submitted to Foundation Medicine Incorporated (FMI). While the value of this data lies in the remarkable size of the dataset, curated clinical data would remain the gold standard. Finally, the study of HRD requires clinical response data to relevant therapeutic agents to be available. The data presented here cannot predict which LMS tumors may be responsive to any therapy. It is the hope of the authors that this work will provide sufficient preliminary data to support a prospective clinical trial testing therapeutic efficacy, most reasonably PARP inhibition, in a genomically defined subset of LMS.

There remains a significant gap in the molecular understanding of LMS. The development of precision therapy for this rare and aggressive disease requires further dissection of molecular subtypes present. Data from our large-scale genomic analysis of LMS suggest that gLOH in LMS differs from other epithelial-derived carcinomas. Correlative data from prospective trials of PARP inhibition in LMS may provide further evidence of HRD as a predictive biomarker in this disease (NCT03880019). Additional studies will be required to validate the use of gLOH as a clinical biomarker in LMS and further unpack the molecular complexities of this disease.

## Methods

### Comprehensive Genomic Profiling

Comprehensive genomic profiling data from LMS tumors were assayed in the course of clinical care using FMI hybrid-capture-based next-generation sequencing platform was provided as previously described^9,38–41^. Approval for the retrospective collection of genomic data from FMI, including a waiver of informed consent and a HIPAA waiver of authorization, was obtained from the Western Institutional Review Board (institutional review protocol number 20152817). From this database, microsatellite status, tumor mutation burden, gLOH, and pathogenicity of genomic alterations were determined utilizing FMI’s analysis pipeline. Only genomic alterations known or likely to be pathogenic were included in this analysis. Variants of unknown significance were excluded from this analysis.

### Development of a HRD related genes

To develop a set of HRD genes for analysis in this study, we performed a comprehensive literature search using PubMed. For the purpose of this study, we defined genes in the HR pathway based on previous reports of correlation with gLOH and clinical response to PARP inhibition across cancer. Additionally, we incorporated HR pathway genes reported to be altered in LMS. A full list of genes included is available in Supplementary Table 2^6,8–11,19,25,42–55^.

### Calculation of percent gLOH

Percent gLOH was calculated as a signature of HRD as previously described^22^. Briefly, LOH segments were inferred across the 22 autosomal chromosomes using the genome-wide aneuploidy/copy number profile and minor allele frequencies of >3,500 polymorphic single nucleotide polymorphisms (SNPs) sequenced in the FoundationOne^®^ assay. Using a comparative genomic hybridization-like method, we obtained a log-ratio profile of the sample by normalizing the sequence coverage obtained at all exons and genome-wide SNPs against a process-matched normal control^38^. This profile was segmented and interpreted using allele frequencies of sequenced SNPs to estimate copy number (Ci) and minor allele count (Mi) at each segment. A segment was determined to have LOH if Ci ≠ 0 and Mi = 0. Low tumor content or low aneuploidy were the most common reasons for failure to pass the quality control to perform gLOH inference. Two types of LOH segments were excluded from the calculation of percent gLOH: LOH segments that spanned ≥90% of a whole chromosome or chromosome arm because these LOH events usually arise through non-HRD mechanisms and regions in which LOH inference was ambiguous. For each tumor, the percent gLOH was computed as 100× the total length of non-excluded LOH regions divided by the total length of non-excluded regions of the genome.

### Retrospective clinical analysis—OSUCCC

Patients with early stage LMS treated at OSUCCC, whose tumors were sequenced by FMI, were identified from the Sarcoma Registry (institutional review protocol number OSU- 2014C0181). Clinical and genomic factors were extracted. All participants identified were included. The sample size was based on data available, and no sample size calculations were performed.

### Statistical methods

All data were analyzed in R_v.3.4.3_ or Graphpad Prism_v.8.0.0_. Two-sided Student’s *t* test, one-way ANOVA, and Chi-squared tests were used, as appropriate. Following use of the one-way ANOVA test, Dunnett’s multiple comparisons test was used to adjust for multiple comparison testing in comparing means to a control group (i.e. no genomic alteration) while Tukey’s multiple comparisons test was used to adjust for multiple comparison testing between means of each group in the model. Continuous data are presented as mean ± SD unless otherwise stated. Unless otherwise stated, *p*-values ≤0.05 were considered statistically significant. Bonferroni adjustment was used to compute *q*-values.

To identify the inflection point in gLOH in this dataset we took the first and second derivatives of the ranked gLOH. The inflection point was defined as the point where the first derivative was greatest while the second derivative was 0. To compare the genomic landscape between uLMS and non-uLMS, we tested the differential prevalence of mutations and copy number variants. A threshold of significance was established as an absolute difference in prevalence between uLMS and non-uLMS of >10% and a p-value of <0.00005 by chi-squared test reflecting a Bonferroni adjustment for multiple comparisons of <0.05. All statistical tests were conducted as two-sided as appropriate. Network analysis was conducted using HumanBase tissue-specific gene networks (hb.flatironinstitute.org, GIANT) using smooth muscle as tissue of origin, a minimum interaction confidence of 0.05, and a maximum additional number of genes of zero^56^. Survival analysis was tested using the log-rank test and Cox proportional hazard regression as appropriate. Survival plots were created using the Kaplan–Meier estimator.

### Reporting summary

Further information on research design is available in the Nature Research Reporting Summary linked to this article.

## Supplementary information

SUPPLEMENTAL MATERIAL

REPORTING SUMMARY

## Data Availability

The data generated and analyzed during this study are described in the following data record: 10.6084/m9.figshare.19208967.v1^57^. This data record also contains data files showing the genomic profiling of pathogenic variants included in this study. Clinical and genomic data from the OSUCCC dataset cannot be openly shared in order to protect patient confidentiality, but can be made available on reasonable request from James L. Chen (James.Chen@osumc.edu).

## Code availability

No custom code was used to complete this analysis.
