# Supplementary material for: Drivers of genomic loss of heterozygosity in leiomyosarcoma are distinct from carcinomas
Source: NPJ Precis Oncol. 2022 Apr 25;6:29. doi: 10.1038/s41698-022-00271-x (PMC9038792; doi:10.1038/s41698-022-00271-x)
Supplement: Supplementary file 1 — SUPPLEMENTAL MATERIAL [file 41698_2022_271_MOESM1_ESM.pdf]

Supplemental Files

Supplemental Figure 1

Supplemental Figure 2

Supplemental Figure 3

Supplemental Table 1

Supplemental Table 2

Supplemental Table 3

Supplemental Table 4

Supplemental Table 5

Supplemental Table 6

Supplementary Figure 1

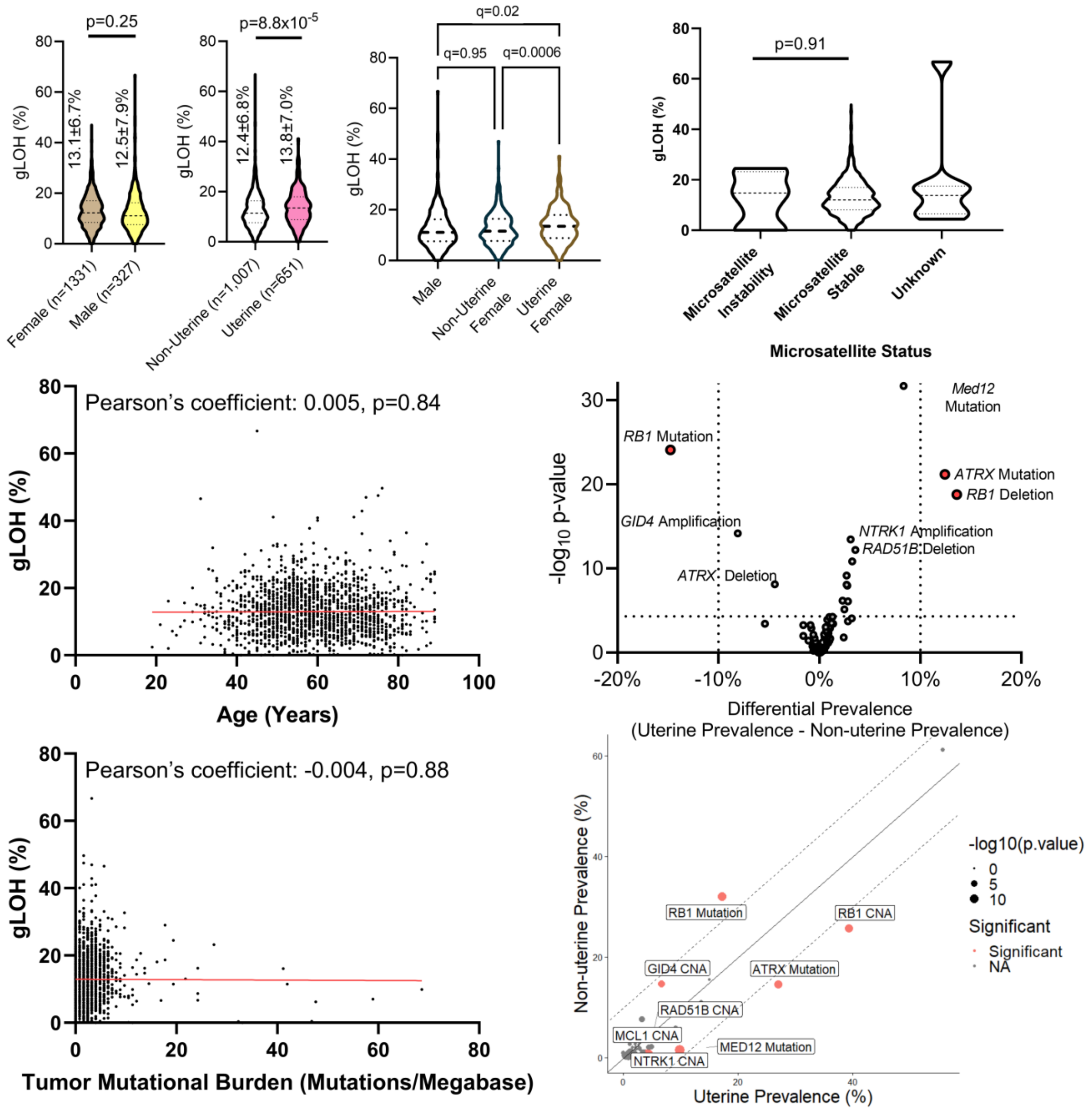

a) gLOH was not correlated with patient sex (Mean±SD; Female n=1,331, 13.1±6.7%; Male n=327, 12.5±7.9%; welch's t-test p=0.25). **b**) gLOH was correlated with LMS of uterine origin (Mean±SD; uLMS n=651, 13.8±7.0%; Non-uLMS n=1,007, 12.4±6.8%; welch's t-test p=8.8x10^{-5}). **c**) No difference in LOH was found between non-uLMS in females and males (Mean±SD; Non-uLMS Female n=680, 12.4±6.5%; Male: 12.5±7.9%; Tukey's multiple comparisons test adjusted q=0.95). uLMS as associated with an increased gLOH compared with non-uLMS in females (Mean±SD; uLMS Female n=651, 13.7±6.9%; Non-uLMS Female n=680, 12.4±6.5%; q=0.0006) and non-uLMS Males (q=0.02). **d-f**) No association was identified between age at sequencing (Pearson's coefficient: 0.005, p=0.84; **d**), tumor mutational burden (Pearson's coefficient: -0.004, p=0.88, **e**), or microsatellite status (Mean±SD, Microsatellite Stable, n=1,643, 12.9±6.9%; Microsatellite Instability, n=7, 12.5±10.0%; welch's t-test p=0.91, **f**) and gLOH in this dataset. **g-h**) Comparison of the genomic landscape of LMS by uterine subtype identified three genes as differentially altered (absolute difference in prevalence between subtypes >10% and p-value <0.00005 [Bonferroni adjustment threshold] by chi-squared test, noted in red). ULMS exhibited an increased prevalence of *ATRX* mutations (uLMS 27.0%, Non-uLMS 14.6%, p=6.4x10^{-10}) and *RB1* homozygous deletion (uLMS 39.3%, Non-uterine 25.7%, p=6.9x10^{-9}). Non-uLMS demonstrated an increased prevalence of *RB1* mutations (uLMS 17.2%, Non-uLMS 31.9%, p=3.5x10^{-11}).

Supplementary Figure 2

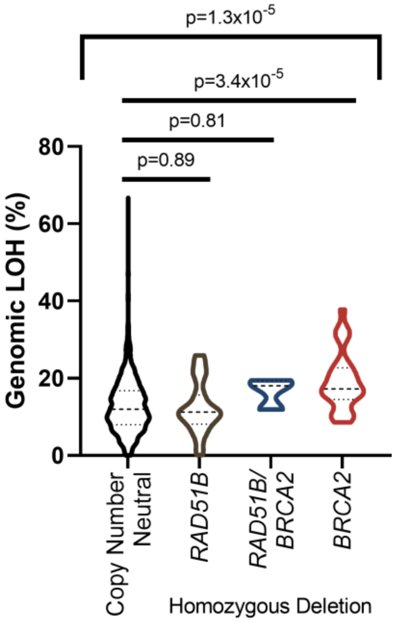

The two most common Homologous Recombination pathway variants, homozygous deletion of *RAD51B* and *BRCA2*, were tested for the effect of their interaction with gLOH. (Mean±SD; Copy number neutral n=1,589, 12.8±6.9%; *RAD51B* Homozygous Deletion n=35, 12.4±6.6%, dunnett's multiple comparisons test vs copy number neutral adjusted q=0.89; *RAD51B*/*BRCA2* Homozygous Deletion n=3, 16.5±3.4%, dunnett's multiple comparisons test vs copy number neutral adjusted q=0.81; *BRCA2* Homozygous Deletion n=31, 19.2±7.4%, dunnett's multiple comparisons test vs copy number neutral adjusted q=$3.4x10^{-5}$).

Supplementary Figure 3

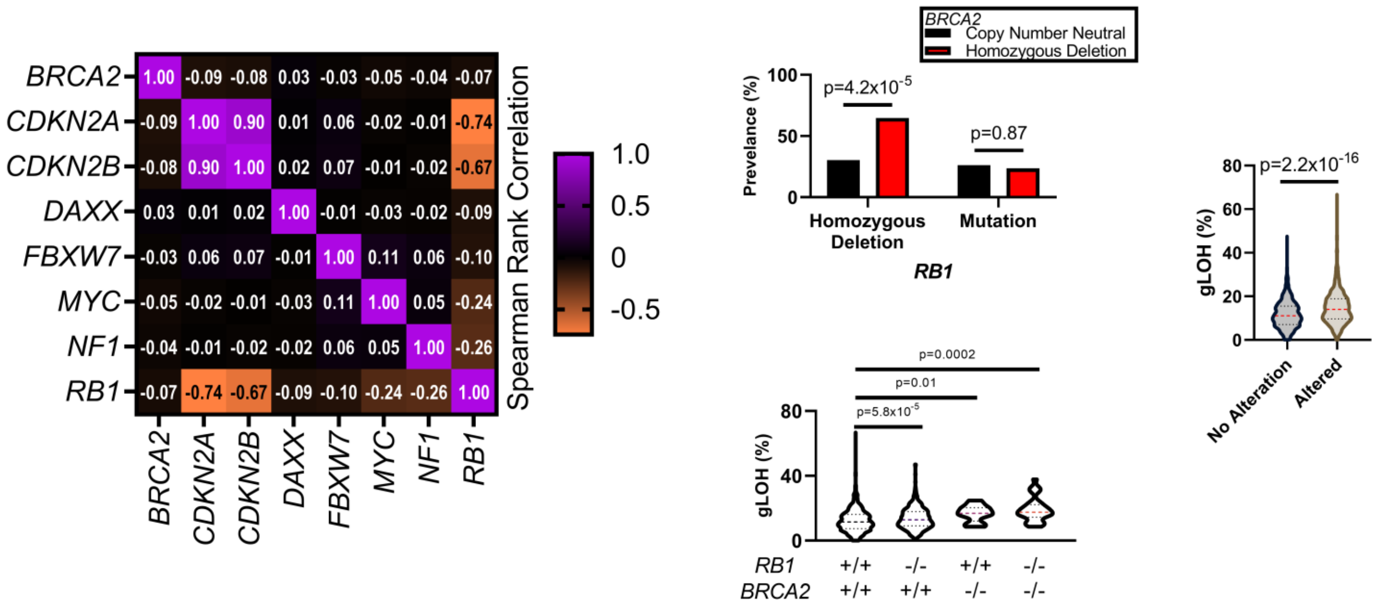

a) Correlation between each of the twelve genomic alterations associate with increased genomic loss of heterozygosity (gLOH) identified mutual exclusivity between *CDKN2A/B* and *RB1* (spearman rank correlation: -0.74 and -0.67), between *NF1* and *RB1* (spearman rank correlation: -0.26), and between *MYC* and *RB1* (spearman rank correlation: -0.24). **b**) Homozygous deletion of *BRCA2* was correlated with an increase prevalence of *RB1* Homozygous deletion (64.7% vs 30.4%; chi-squared $p=4.2x10^{-5}$), but was not associated with a difference in prevalence of *RB1* mutations (23.5% vs 26.2%; chi-squared p=0.87). **c**) Homozygous deletion of either *RB1* or *BRCA2* was associated with increased gLOH. Presence of homozygous deletion both *RB1* and *BRCA2* was associated with the greatest increase of gLOH (Mean±SD, p-value by welch's t-test comparison to intact samples; Intact [n=1,143] 12.7±7.0%; *RB1* [n=493] 13.8±6.6%, $p=5.8x10^{-5}$; *BRCA2* homozygous deletion alone [n=12] 16.7±5.1%, p=0.01; *RB1* and *BRCA2* homozygous deletion [n=34] 18.9±7.2%, p<0.0001). **d**) The presence of any of the eight genomic alterations identified was associated in an increased gLOH (No alteration: n=989, 11.7±6.3%; Alteration: n=669, 14.8±7.5%; $p=2.2x10^{-16}$).

Supplementary Table 1: genomic alterations between uterine and non-uterine LMS

Supplementary Table 1: genomic alterations between uterine and non-uterine LMS				
Alteration	Uterine Prevalence	Non-uterine Prevalence	Differential Prevalence	p Value
MED12 Mutation	9.83%	1.49%	8.34%	1.74E-14
RB1 Mutation	17.20%	31.98%	-14.77%	3.48E-11
ATRX Mutation	27.04%	14.60%	12.44%	6.37E-10
RB1 CNA	39.32%	25.72%	13.60%	6.98E-09
GID4 CNA	6.61%	14.70%	-8.09%	7.01E-07
NTRK1 CNA	3.38%	0.30%	3.08%	1.42E-06
RAD51B CNA	4.45%	0.89%	3.56%	5.03E-06
MCL1 CNA	4.15%	0.89%	3.25%	1.98E-05
DAXX Mutation	3.38%	0.70%	2.68%	0.000105
ATRX CNA	3.23%	7.65%	-4.42%	0.000294
BRCA2 CNA	3.69%	0.99%	2.69%	0.000316
CKS1B CNA	3.84%	1.09%	2.75%	0.000348
ALK Mutation	3.38%	1.09%	2.29%	0.002098
CDKN2C CNA	4.92%	2.09%	2.83%	0.002242
FAF1 CNA	4.45%	1.99%	2.47%	0.00596
FOXP1 CNA	1.69%	0.40%	1.29%	0.014336
PIK3R1 CNA	1.08%	0.10%	0.98%	0.014782
CDKN2A CNA	9.06%	5.86%	3.20%	0.017307
PIK3CA CNA	0.77%	0.00%	0.77%	0.019983
CDKN2B CNA	7.68%	4.87%	2.81%	0.024083
KIT CNA	1.69%	0.50%	1.19%	0.030028
PDGFRA CNA	1.84%	0.60%	1.25%	0.03147
TP53 Mutation	55.76%	61.17%	-5.41%	0.032594
BRCA2 Mutation	2.15%	0.79%	1.36%	0.032611
AKT2 CNA	0.00%	0.89%	-0.89%	0.037851
IGF1R CNA	1.08%	2.68%	-1.61%	0.037914
LRP1B CNA	0.61%	0.00%	0.61%	0.04794
BRAF CNA	0.61%	0.00%	0.61%	0.04794
TBL1XR1 CNA	0.61%	0.00%	0.61%	0.04794
TAF1 Mutation	0.61%	0.00%	0.61%	0.04794
PDGFRA Mutation	0.61%	0.00%	0.61%	0.04794
FGF6 CNA	1.23%	0.30%	0.93%	0.048777
KDR CNA	1.54%	0.50%	1.04%	0.055161
AXL CNA	0.00%	0.79%	-0.79%	0.055264
ESR1 Mutation	0.77%	0.10%	0.67%	0.072534
LPP Mutation	0.77%	0.10%	0.67%	0.072534
NCOR1 CNA	0.92%	0.20%	0.72%	0.086908
TOP1 CNA	1.08%	0.30%	0.78%	0.094604
PIK3R1 Mutation	1.08%	0.30%	0.78%	0.094604

CIITA Mutation	1.08%	0.30%	0.78%	0.094604
CCND2 CNA	1.23%	0.40%	0.83%	0.098073
FGF23 CNA	1.23%	0.40%	0.83%	0.098073
NOTCH3 CNA	1.23%	0.40%	0.83%	0.098073
NOTCH4 Mutation	1.23%	0.40%	0.83%	0.098073
ATR Mutation	1.38%	0.50%	0.89%	0.098842
MAF CNA	0.46%	0.00%	0.46%	0.117707
ZNF217 CNA	0.46%	0.00%	0.46%	0.117707
SH2B3 Mutation	0.46%	0.00%	0.46%	0.117707
BLM Mutation	0.46%	0.00%	0.46%	0.117707
TLL2 Mutation	0.46%	0.00%	0.46%	0.117707
HSP90AA1 Mutation	0.46%	0.00%	0.46%	0.117707
ETV6 Mutation	0.00%	0.60%	-0.60%	0.120114
AURKB CNA	3.07%	4.67%	-1.60%	0.138066
PTPRO Mutation	0.61%	0.10%	0.52%	0.158687
FBXW7 Mutation	0.61%	0.10%	0.52%	0.158687
ESR1 CNA	1.38%	0.60%	0.79%	0.165604
TSC2 Mutation	1.38%	0.60%	0.79%	0.165604
PTEN CNA	13.52%	11.12%	2.40%	0.165955
STK11 Mutation	0.77%	0.20%	0.57%	0.174304
BCOR Mutation	0.77%	0.20%	0.57%	0.174304
PRDM1 Mutation	0.77%	0.20%	0.57%	0.174304
MITF CNA	1.08%	0.40%	0.68%	0.176679
DAXX CNA	0.92%	0.30%	0.62%	0.178374
BCL2 CNA	0.92%	0.30%	0.62%	0.178374
PDGFRB Mutation	0.92%	0.30%	0.62%	0.178374
SDHA CNA	0.00%	0.50%	-0.50%	0.179592
FAF1 Mutation	0.00%	0.50%	-0.50%	0.179592
CDK4 CNA	2.76%	1.69%	1.08%	0.188656
RICTOR CNA	2.00%	3.08%	-1.08%	0.237384
FAS CNA	2.76%	1.79%	0.98%	0.245604
PCLO Mutation	0.00%	0.40%	-0.40%	0.272442
IRS2 CNA	1.84%	1.09%	0.75%	0.288361
MTOR CNA	0.31%	0.00%	0.31%	0.300424
BARD1 CNA	0.31%	0.00%	0.31%	0.300424
CPS1 CNA	0.31%	0.00%	0.31%	0.300424
FBXO11 CNA	0.31%	0.00%	0.31%	0.300424
MSH6 CNA	0.31%	0.00%	0.31%	0.300424
SDHB CNA	0.31%	0.00%	0.31%	0.300424
ETV6 CNA	0.31%	0.00%	0.31%	0.300424
ACTB Mutation	0.31%	0.00%	0.31%	0.300424
BARD1 Mutation	0.31%	0.00%	0.31%	0.300424
FAS Mutation	0.31%	0.00%	0.31%	0.300424

SDHC Mutation	0.31%	0.00%	0.31%	0.300424
SMARCD1 Mutation	0.31%	0.00%	0.31%	0.300424
SMARCB1 Mutation	0.31%	0.00%	0.31%	0.300424
FANCG Mutation	0.31%	0.00%	0.31%	0.300424
PDGFB Mutation	0.31%	0.00%	0.31%	0.300424
TMEM30A Mutation	0.31%	0.00%	0.31%	0.300424
HMGA2 Mutation	0.92%	0.40%	0.52%	0.306746
CCNE1 CNA	2.76%	1.89%	0.88%	0.311555
CDKN1B CNA	0.77%	0.30%	0.47%	0.324039
NOD1 Mutation	0.77%	0.30%	0.47%	0.324039
VHL Mutation	0.15%	0.60%	-0.44%	0.332867
ERBB3 CNA	0.61%	0.20%	0.42%	0.337942
EED CNA	0.61%	0.20%	0.42%	0.337942
CCND1 CNA	0.61%	0.20%	0.42%	0.337942
SUFU Mutation	0.61%	0.20%	0.42%	0.337942
PBRM1 Mutation	0.61%	0.20%	0.42%	0.337942
CTNNB1 Mutation	0.61%	0.20%	0.42%	0.337942
FANCA CNA	0.46%	0.10%	0.36%	0.340702
KDM6A CNA	0.46%	0.10%	0.36%	0.340702
TSC1 CNA	0.46%	0.10%	0.36%	0.340702
CAD Mutation	0.46%	0.10%	0.36%	0.340702
EZH2 Mutation	0.46%	0.10%	0.36%	0.340702
BRSK1 Mutation	0.46%	0.10%	0.36%	0.340702
CBL Mutation	0.46%	0.10%	0.36%	0.340702
PAX5 Mutation	0.46%	0.10%	0.36%	0.340702
B2M Mutation	0.46%	0.10%	0.36%	0.340702
NOTCH2 Mutation	1.38%	0.79%	0.59%	0.36223
NOTCH3 Mutation	1.23%	0.70%	0.53%	0.392368
TSC1 Mutation	1.23%	0.70%	0.53%	0.392368
CUX1 Mutation	1.69%	1.09%	0.60%	0.413177
STAG2 CNA	0.00%	0.30%	-0.30%	0.422425
NSD1 Mutation	0.00%	0.30%	-0.30%	0.422425
AKT1 Mutation	0.00%	0.30%	-0.30%	0.422425
PTPN6 Mutation	0.00%	0.30%	-0.30%	0.422425
EPHA5 Mutation	0.00%	0.30%	-0.30%	0.422425
FAT3 Mutation	0.00%	0.30%	-0.30%	0.422425
NTRK1 Mutation	0.00%	0.30%	-0.30%	0.422425
JAK1 Mutation	0.00%	0.30%	-0.30%	0.422425
RET Mutation	0.00%	0.30%	-0.30%	0.422425
SDHA Mutation	1.08%	0.60%	0.48%	0.426157
BRD4 Mutation	1.08%	0.60%	0.48%	0.426157
FGF14 CNA	0.77%	1.29%	-0.52%	0.446814
CHEK2 Mutation	0.77%	1.29%	-0.52%	0.446814

CREBBP Mutation	0.77%	1.29%	-0.52%	0.446814
EPHA3 CNA	0.92%	0.50%	0.43%	0.46443
KMT2D Mutation	2.00%	2.68%	-0.68%	0.469726
ARAF CNA	0.15%	0.50%	-0.34%	0.473507
TCF3 Mutation	0.15%	0.50%	-0.34%	0.473507
ICK Mutation	0.15%	0.50%	-0.34%	0.473507
RAD54L Mutation	0.15%	0.50%	-0.34%	0.473507
CD274 CNA	0.31%	0.70%	-0.39%	0.479216
PDCD1LG2 CNA	0.31%	0.70%	-0.39%	0.479216
MSH2 Mutation	0.31%	0.70%	-0.39%	0.479216
EP300 Mutation	0.31%	0.70%	-0.39%	0.479216
CD36 Mutation	2.30%	1.69%	0.62%	0.479261
NCOR1 Mutation	1.38%	0.89%	0.49%	0.486947
PASK Mutation	1.38%	0.89%	0.49%	0.486947
SUFU CNA	0.77%	0.40%	0.37%	0.508401
MEN1 Mutation	0.77%	0.40%	0.37%	0.508401
APC Mutation	0.92%	1.39%	-0.47%	0.533137
LRP1B Mutation	2.00%	1.49%	0.51%	0.556664
FGF3 CNA	0.61%	0.30%	0.32%	0.559971
NKX2-1 CNA	0.61%	0.30%	0.32%	0.559971
NFKBIA CNA	0.61%	0.30%	0.32%	0.559971
FGF4 CNA	0.61%	0.30%	0.32%	0.559971
FGF19 CNA	0.61%	0.30%	0.32%	0.559971
FOXP1 Mutation	0.61%	0.30%	0.32%	0.559971
ARID1B Mutation	0.61%	0.30%	0.32%	0.559971
CPS1 Mutation	0.61%	0.30%	0.32%	0.559971
FANCD2 Mutation	0.61%	0.30%	0.32%	0.559971
CCND3 CNA	0.61%	0.99%	-0.38%	0.583723
CRKL CNA	1.08%	1.49%	-0.41%	0.616924
RPTOR CNA	0.46%	0.20%	0.26%	0.622487
DNMT3A CNA	0.46%	0.20%	0.26%	0.622487
MDM4 CNA	0.46%	0.20%	0.26%	0.622487
FHIT Mutation	0.46%	0.20%	0.26%	0.622487
BACH1 Mutation	0.46%	0.20%	0.26%	0.622487
KRAS Mutation	0.46%	0.20%	0.26%	0.622487
NUP98 Mutation	0.46%	0.20%	0.26%	0.622487
AMER1 Mutation	0.46%	0.20%	0.26%	0.622487
WT1 Mutation	0.46%	0.20%	0.26%	0.622487
RUNX1 Mutation	0.46%	0.20%	0.26%	0.622487
POT1 Mutation	0.46%	0.20%	0.26%	0.622487
PIK3C2G Mutation	0.46%	0.20%	0.26%	0.622487
FGF10 CNA	1.54%	1.99%	-0.45%	0.629338
DNMT3A Mutation	1.54%	1.99%	-0.45%	0.629338

JAK2 CNA	0.31%	0.60%	-0.29%	0.641716
PIM1 CNA	0.31%	0.60%	-0.29%	0.641716
BIRC3 CNA	0.31%	0.60%	-0.29%	0.641716
GNAS CNA	0.15%	0.40%	-0.24%	0.670952
CBFB CNA	0.15%	0.40%	-0.24%	0.670952
MSH3 Mutation	0.15%	0.40%	-0.24%	0.670952
ZRSR2 Mutation	0.15%	0.40%	-0.24%	0.670952
CDC73 Mutation	0.15%	0.40%	-0.24%	0.670952
MAP2K1 CNA	0.00%	0.20%	-0.20%	0.679361
INPP4B CNA	0.00%	0.20%	-0.20%	0.679361
NRAS CNA	0.00%	0.20%	-0.20%	0.679361
MEF2C CNA	0.00%	0.20%	-0.20%	0.679361
CD58 CNA	0.00%	0.20%	-0.20%	0.679361
IGF2 CNA	0.00%	0.20%	-0.20%	0.679361
HRAS CNA	0.00%	0.20%	-0.20%	0.679361
LRRK2 Mutation	0.00%	0.20%	-0.20%	0.679361
MET Mutation	0.00%	0.20%	-0.20%	0.679361
PLCG2 Mutation	0.00%	0.20%	-0.20%	0.679361
BCL10 Mutation	0.00%	0.20%	-0.20%	0.679361
TYK2 Mutation	0.00%	0.20%	-0.20%	0.679361
FGFR3 Mutation	0.00%	0.20%	-0.20%	0.679361
TNFAIP3 Mutation	0.00%	0.20%	-0.20%	0.679361
TRAF2 Mutation	0.00%	0.20%	-0.20%	0.679361
STAT6 Mutation	0.00%	0.20%	-0.20%	0.679361
RARA Mutation	0.00%	0.20%	-0.20%	0.679361
BAP1 Mutation	0.00%	0.20%	-0.20%	0.679361
TRAF3 Mutation	0.00%	0.20%	-0.20%	0.679361
MAP3K1 Mutation	0.00%	0.20%	-0.20%	0.679361
IKZF1 Mutation	0.00%	0.20%	-0.20%	0.679361
STAT4 Mutation	0.00%	0.20%	-0.20%	0.679361
PAK5 Mutation	0.00%	0.20%	-0.20%	0.679361
MAF Mutation	0.00%	0.20%	-0.20%	0.679361
NFKBIA Mutation	0.00%	0.20%	-0.20%	0.679361
INPP4B Mutation	0.00%	0.20%	-0.20%	0.679361
MAP3K13 Mutation	0.00%	0.20%	-0.20%	0.679361
SETBP1 Mutation	0.00%	0.20%	-0.20%	0.679361
BIRC3 Mutation	0.00%	0.20%	-0.20%	0.679361
CBFB Mutation	0.00%	0.20%	-0.20%	0.679361
MTAP Mutation	0.00%	0.20%	-0.20%	0.679361
CDKN1B Mutation	0.00%	0.20%	-0.20%	0.679361
EWSR1 Mutation	0.00%	0.20%	-0.20%	0.679361
RNF213 Mutation	0.00%	0.20%	-0.20%	0.679361
HLA-A Mutation	0.00%	0.20%	-0.20%	0.679361

KMT2C Mutation	2.61%	2.18%	0.43%	0.693675
MAP3K6 Mutation	1.23%	1.59%	-0.36%	0.697421
ERBB2 CNA	0.31%	0.10%	0.21%	0.70311
PBRM1 CNA	0.31%	0.10%	0.21%	0.70311
MALT1 CNA	0.31%	0.10%	0.21%	0.70311
BAP1 CNA	0.31%	0.10%	0.21%	0.70311
NF2 CNA	0.31%	0.10%	0.21%	0.70311
SRC CNA	0.31%	0.10%	0.21%	0.70311
MLH1 CNA	0.31%	0.10%	0.21%	0.70311
ABL1 Mutation	0.31%	0.10%	0.21%	0.70311
TRAF5 Mutation	0.31%	0.10%	0.21%	0.70311
HNF1A Mutation	0.31%	0.10%	0.21%	0.70311
EPHA3 Mutation	0.31%	0.10%	0.21%	0.70311
APCDD1 Mutation	0.31%	0.10%	0.21%	0.70311
AKT3 Mutation	0.31%	0.10%	0.21%	0.70311
SS18 Mutation	0.31%	0.10%	0.21%	0.70311
ARID1A Mutation	1.08%	0.79%	0.28%	0.745792
PTEN Mutation	5.84%	5.36%	0.47%	0.762283
NF1 CNA	1.38%	1.09%	0.29%	0.765599
TET2 Mutation	1.38%	1.09%	0.29%	0.765599
MYC CNA	2.00%	1.69%	0.31%	0.785671
FRS2 CNA	2.00%	1.69%	0.31%	0.785671
PTCH1 Mutation	0.61%	0.40%	0.22%	0.79452
TP53 CNA	14.90%	15.49%	-0.59%	0.797101
BCL2L2 CNA	0.92%	0.70%	0.23%	0.821514
NCOR2 Mutation	0.92%	0.70%	0.23%	0.821514
GADD45B CNA	0.15%	0.00%	0.15%	0.825941
PTPRO CNA	0.15%	0.00%	0.15%	0.825941
SMARCA4 CNA	0.15%	0.00%	0.15%	0.825941
FLT4 CNA	0.15%	0.00%	0.15%	0.825941
CUX1 CNA	0.15%	0.00%	0.15%	0.825941
BRCA1 CNA	0.15%	0.00%	0.15%	0.825941
ATM CNA	0.15%	0.00%	0.15%	0.825941
IKZF3 CNA	0.15%	0.00%	0.15%	0.825941
FBXO31 CNA	0.15%	0.00%	0.15%	0.825941
PASK CNA	0.15%	0.00%	0.15%	0.825941
CREBBP CNA	0.15%	0.00%	0.15%	0.825941
STAT4 CNA	0.15%	0.00%	0.15%	0.825941
RAF1 CNA	0.15%	0.00%	0.15%	0.825941
CASP8 CNA	0.15%	0.00%	0.15%	0.825941
SUZ12 CNA	0.15%	0.00%	0.15%	0.825941
ALK CNA	0.15%	0.00%	0.15%	0.825941
RUNX1 CNA	0.15%	0.00%	0.15%	0.825941

PAX5 CNA	0.15%	0.00%	0.15%	0.825941
PHF6 CNA	0.15%	0.00%	0.15%	0.825941
PRSS8 CNA	0.15%	0.00%	0.15%	0.825941
AURKA CNA	0.15%	0.00%	0.15%	0.825941
NTRK2 Mutation	0.15%	0.00%	0.15%	0.825941
FLT4 Mutation	0.15%	0.00%	0.15%	0.825941
CARD11 Mutation	0.15%	0.00%	0.15%	0.825941
IDH2 Mutation	0.15%	0.00%	0.15%	0.825941
IGF1R Mutation	0.15%	0.00%	0.15%	0.825941
IKZF3 Mutation	0.15%	0.00%	0.15%	0.825941
DDX3X Mutation	0.15%	0.00%	0.15%	0.825941
FBXO31 Mutation	0.15%	0.00%	0.15%	0.825941
KDR Mutation	0.15%	0.00%	0.15%	0.825941
FLT1 Mutation	0.15%	0.00%	0.15%	0.825941
CD274 Mutation	0.15%	0.00%	0.15%	0.825941
GNAS Mutation	0.15%	0.00%	0.15%	0.825941
AXL Mutation	0.15%	0.00%	0.15%	0.825941
BCL11B Mutation	0.15%	0.00%	0.15%	0.825941
GATA1 Mutation	0.15%	0.00%	0.15%	0.825941
PDCD11 Mutation	0.15%	0.00%	0.15%	0.825941
SOX10 Mutation	0.15%	0.00%	0.15%	0.825941
PRKDC Mutation	0.15%	0.00%	0.15%	0.825941
FGFR2 Mutation	0.15%	0.00%	0.15%	0.825941
SMAD4 Mutation	0.15%	0.00%	0.15%	0.825941
TBX3 Mutation	0.15%	0.00%	0.15%	0.825941
DUSP9 Mutation	0.15%	0.00%	0.15%	0.825941
CASP8 Mutation	0.15%	0.00%	0.15%	0.825941
NFE2L2 Mutation	0.15%	0.00%	0.15%	0.825941
NSD2 Mutation	0.15%	0.00%	0.15%	0.825941
U2AF1 Mutation	0.15%	0.00%	0.15%	0.825941
FLT3 Mutation	0.15%	0.00%	0.15%	0.825941
FGF14 Mutation	0.15%	0.00%	0.15%	0.825941
INPP5D Mutation	0.15%	0.00%	0.15%	0.825941
DTX1 Mutation	0.15%	0.00%	0.15%	0.825941
CCND1 Mutation	0.15%	0.00%	0.15%	0.825941
PTPN2 Mutation	0.15%	0.00%	0.15%	0.825941
NRAS Mutation	0.15%	0.00%	0.15%	0.825941
MYCN Mutation	0.15%	0.00%	0.15%	0.825941
CD58 Mutation	0.15%	0.00%	0.15%	0.825941
MLH1 Mutation	0.15%	0.00%	0.15%	0.825941
TGFBR2 Mutation	0.15%	0.00%	0.15%	0.825941
GAS7 Mutation	0.15%	0.00%	0.15%	0.825941
RAD51D Mutation	0.15%	0.00%	0.15%	0.825941

NBEAP1 Mutation	0.15%	0.00%	0.15%	0.825941
TRIM24 Mutation	0.15%	0.00%	0.15%	0.825941
BCL3 Mutation	0.15%	0.00%	0.15%	0.825941
AR CNA	0.31%	0.50%	-0.19%	0.847166
FANCA Mutation	0.31%	0.50%	-0.19%	0.847166
ZNF703 CNA	1.38%	1.59%	-0.21%	0.896232
EGFR CNA	0.61%	0.79%	-0.18%	0.900049
KDM5A CNA	0.61%	0.79%	-0.18%	0.900049
ATM Mutation	0.61%	0.79%	-0.18%	0.900049
STK11 CNA	0.46%	0.30%	0.16%	0.903907
MET CNA	0.46%	0.30%	0.16%	0.903907
TNFRSF11A CNA	0.46%	0.30%	0.16%	0.903907
BRCA1 Mutation	0.46%	0.30%	0.16%	0.903907
HDAC4 Mutation	0.46%	0.30%	0.16%	0.903907
PIK3CA Mutation	1.38%	1.19%	0.19%	0.908871
CUL4A CNA	2.76%	2.98%	-0.21%	0.917153
MAPK1 CNA	0.92%	1.09%	-0.17%	0.930418
CHEK2 CNA	0.15%	0.30%	-0.14%	0.942332
MKI67 Mutation	0.15%	0.30%	-0.14%	0.942332
SPEN Mutation	0.15%	0.30%	-0.14%	0.942332
CHD2 Mutation	0.15%	0.30%	-0.14%	0.942332
TBL1XR1 Mutation	0.15%	0.30%	-0.14%	0.942332
MTOR Mutation	0.15%	0.30%	-0.14%	0.942332
PIK3R2 Mutation	0.15%	0.30%	-0.14%	0.942332
JARID2 Mutation	0.15%	0.30%	-0.14%	0.942332
PICALM Mutation	0.15%	0.30%	-0.14%	0.942332
CDKN2A Mutation	1.23%	1.39%	-0.16%	0.951595
CIC CNA	1.54%	1.39%	0.15%	0.974271
STAG2 Mutation	0.46%	0.60%	-0.13%	0.981556
RAD50 Mutation	0.46%	0.60%	-0.13%	0.981556
CCT6B Mutation	1.23%	1.09%	0.14%	0.984994
MUTYH Mutation	2.15%	2.28%	-0.13%	0.992463
FHIT CNA	0.15%	0.10%	0.05%	1
ROS1 CNA	0.15%	0.10%	0.05%	1
AKT1 CNA	0.15%	0.10%	0.05%	1
NCOR2 CNA	0.15%	0.10%	0.05%	1
TSC2 CNA	0.15%	0.10%	0.05%	1
BCORL1 CNA	0.15%	0.10%	0.05%	1
IKBKE CNA	0.15%	0.10%	0.05%	1
PMS2 CNA	0.15%	0.10%	0.05%	1
PIK3CG CNA	0.15%	0.10%	0.05%	1
REL CNA	0.15%	0.10%	0.05%	1
RET CNA	0.15%	0.10%	0.05%	1

CDK6 CNA	0.15%	0.10%	0.05%	1
KAT6A Mutation	0.15%	0.10%	0.05%	1
FANCM Mutation	0.15%	0.10%	0.05%	1
ARHGAP26 Mutation	0.15%	0.10%	0.05%	1
CXCR4 Mutation	0.15%	0.10%	0.05%	1
NTRK3 Mutation	0.15%	0.10%	0.05%	1
ERBB3 Mutation	0.15%	0.10%	0.05%	1
MAGED1 Mutation	0.15%	0.10%	0.05%	1
CHUK Mutation	0.15%	0.10%	0.05%	1
KDM5C Mutation	0.15%	0.10%	0.05%	1
CDKN2B Mutation	0.15%	0.10%	0.05%	1
CDKN2C Mutation	0.15%	0.10%	0.05%	1
JAK2 Mutation	0.15%	0.10%	0.05%	1
PARP4 Mutation	0.15%	0.10%	0.05%	1
HGF Mutation	0.15%	0.10%	0.05%	1
KIT Mutation	0.15%	0.10%	0.05%	1
MALT1 Mutation	0.15%	0.10%	0.05%	1
RASGEF1A Mutation	0.15%	0.10%	0.05%	1
RUNX1T1 Mutation	0.15%	0.10%	0.05%	1
PIK3CG Mutation	0.15%	0.10%	0.05%	1
RAF1 Mutation	0.15%	0.10%	0.05%	1
BCL6 Mutation	0.15%	0.10%	0.05%	1
CTCF Mutation	0.15%	0.10%	0.05%	1
FGFR1 Mutation	0.15%	0.10%	0.05%	1
XRCC3 Mutation	0.15%	0.10%	0.05%	1
FANCC Mutation	0.15%	0.10%	0.05%	1
SDHB Mutation	0.15%	0.10%	0.05%	1
FANCE Mutation	0.15%	0.10%	0.05%	1
FGF6 Mutation	0.15%	0.10%	0.05%	1
CCND3 Mutation	0.15%	0.10%	0.05%	1
INHBA Mutation	0.15%	0.10%	0.05%	1
SMO Mutation	0.15%	0.10%	0.05%	1
RAD51C Mutation	0.15%	0.10%	0.05%	1
BCR Mutation	0.15%	0.10%	0.05%	1
PHF6 Mutation	0.15%	0.10%	0.05%	1
FANCL Mutation	0.15%	0.10%	0.05%	1
NCOA2 Mutation	0.15%	0.10%	0.05%	1
ARFRP1 CNA	0.15%	0.20%	-0.04%	1
MSH2 CNA	0.15%	0.20%	-0.04%	1
FGFR4 CNA	0.15%	0.20%	-0.04%	1
MAP2K2 CNA	0.15%	0.20%	-0.04%	1
MYCL CNA	0.15%	0.20%	-0.04%	1
FGF7 CNA	0.15%	0.20%	-0.04%	1

SOX2 CNA	0.15%	0.20%	-0.04%	1
WDR90 Mutation	0.15%	0.20%	-0.04%	1
ROS1 Mutation	0.15%	0.20%	-0.04%	1
PRKAR1A Mutation	0.15%	0.20%	-0.04%	1
CDH1 Mutation	0.15%	0.20%	-0.04%	1
TP63 Mutation	0.15%	0.20%	-0.04%	1
KEAP1 Mutation	0.15%	0.20%	-0.04%	1
BCL7A Mutation	0.15%	0.20%	-0.04%	1
IGH Mutation	0.15%	0.20%	-0.04%	1
PLAG1 Mutation	0.15%	0.20%	-0.04%	1
FLCN Mutation	0.46%	0.50%	-0.04%	1
NOTCH1 Mutation	0.46%	0.50%	-0.04%	1
SETD2 Mutation	0.46%	0.50%	-0.04%	1
ZMYM3 Mutation	0.46%	0.50%	-0.04%	1
PALB2 Mutation	0.46%	0.50%	-0.04%	1
KDM4C CNA	0.46%	0.40%	0.06%	1
MAP2K4 Mutation	0.46%	0.40%	0.06%	1
BCORL1 Mutation	0.46%	0.40%	0.06%	1
ARID2 Mutation	0.46%	0.40%	0.06%	1
KAT6A CNA	1.08%	1.19%	-0.12%	1
FGFR1 CNA	1.38%	1.49%	-0.11%	1
MDM2 CNA	2.00%	1.99%	0.01%	1
NF1 Mutation	2.61%	2.58%	0.03%	1
JUN CNA	1.23%	1.29%	-0.06%	1
SMARCA4 Mutation	1.08%	1.09%	-0.02%	1
BACH1 CNA	0.00%	0.10%	-0.10%	1
EMSY CNA	0.31%	0.20%	0.11%	1
RAD21 CNA	1.54%	1.49%	0.05%	1
ERBB4 CNA	0.00%	0.10%	-0.10%	1
CSF3R CNA	0.00%	0.10%	-0.10%	1
FGFR3 CNA	0.31%	0.40%	-0.09%	1
KRAS CNA	0.61%	0.60%	0.02%	1
TP63 CNA	0.00%	0.10%	-0.10%	1
KMT2C CNA	0.00%	0.10%	-0.10%	1
NOTCH4 CNA	0.00%	0.10%	-0.10%	1
ZRSR2 CNA	0.00%	0.10%	-0.10%	1
FGFR2 CNA	0.31%	0.20%	0.11%	1
SMAD4 CNA	0.00%	0.10%	-0.10%	1
FGF12 CNA	0.31%	0.40%	-0.09%	1
MAP3K14 CNA	0.31%	0.20%	0.11%	1
FLT3 CNA	0.61%	0.50%	0.12%	1
CDK8 CNA	0.61%	0.50%	0.12%	1
HDAC4 CNA	0.31%	0.20%	0.11%	1

PALB2 CNA	0.00%	0.10%	-0.10%	1
TNFRSF14 CNA	0.00%	0.10%	-0.10%	1
PARP1 CNA	0.00%	0.10%	-0.10%	1
GADD45B Mutation	0.00%	0.10%	-0.10%	1
RELN Mutation	0.31%	0.40%	-0.09%	1
EPHB1 Mutation	0.00%	0.10%	-0.10%	1
RAD51B Mutation	0.31%	0.40%	-0.09%	1
AR Mutation	0.00%	0.10%	-0.10%	1
EPHA7 Mutation	0.00%	0.10%	-0.10%	1
FOXO1 Mutation	0.31%	0.40%	-0.09%	1
EGFR Mutation	0.00%	0.10%	-0.10%	1
KDM5A Mutation	0.00%	0.10%	-0.10%	1
RAD21 Mutation	0.31%	0.20%	0.11%	1
SMAD2 Mutation	0.00%	0.10%	-0.10%	1
MRE11 Mutation	0.61%	0.50%	0.12%	1
ERBB4 Mutation	0.00%	0.10%	-0.10%	1
KDM6A Mutation	0.61%	0.60%	0.02%	1
CIC Mutation	0.61%	0.50%	0.12%	1
BRAF Mutation	0.61%	0.50%	0.12%	1
ECT2L Mutation	0.00%	0.10%	-0.10%	1
AXIN1 Mutation	0.31%	0.20%	0.11%	1
ASMTL Mutation	0.00%	0.10%	-0.10%	1
JAK3 Mutation	0.00%	0.10%	-0.10%	1
PTPN11 Mutation	0.00%	0.10%	-0.10%	1
RPTOR Mutation	0.00%	0.10%	-0.10%	1
IDH1 Mutation	0.00%	0.10%	-0.10%	1
SGK1 Mutation	0.00%	0.10%	-0.10%	1
GRIN2A Mutation	0.31%	0.20%	0.11%	1
HDAC1 Mutation	0.00%	0.10%	-0.10%	1
PDCD1LG2 Mutation	0.00%	0.10%	-0.10%	1
DNM2 Mutation	0.31%	0.20%	0.11%	1
NPM1 Mutation	0.00%	0.10%	-0.10%	1
PC Mutation	0.00%	0.10%	-0.10%	1
TUSC3 Mutation	0.00%	0.10%	-0.10%	1
FBXO11 Mutation	0.00%	0.10%	-0.10%	1
PMS2 Mutation	0.31%	0.20%	0.11%	1
LEF1 Mutation	0.00%	0.10%	-0.10%	1
ERG Mutation	0.00%	0.10%	-0.10%	1
FGF23 Mutation	0.00%	0.10%	-0.10%	1
HIST1H1D Mutation	0.00%	0.10%	-0.10%	1
NF2 Mutation	0.61%	0.50%	0.12%	1
FOXL2 Mutation	0.00%	0.10%	-0.10%	1
MAP3K14 Mutation	0.00%	0.10%	-0.10%	1

FGFR4 Mutation	0.00%	0.10%	-0.10%	1
SMC1A Mutation	0.00%	0.10%	-0.10%	1
TOP1 Mutation	0.00%	0.10%	-0.10%	1
RAD51 Mutation	0.00%	0.10%	-0.10%	1
GNAQ Mutation	0.00%	0.10%	-0.10%	1
MAP2K2 Mutation	0.00%	0.10%	-0.10%	1
KMT2A Mutation	0.00%	0.10%	-0.10%	1
ASXL1 Mutation	0.61%	0.50%	0.12%	1
SUZ12 Mutation	0.31%	0.20%	0.11%	1
CTNNA1 Mutation	0.31%	0.20%	0.11%	1
GATA2 Mutation	0.00%	0.10%	-0.10%	1
CEBPA Mutation	0.00%	0.10%	-0.10%	1
PPP2R1A Mutation	0.00%	0.10%	-0.10%	1
CSF1R Mutation	0.00%	0.10%	-0.10%	1
CRLF2 Mutation	0.00%	0.10%	-0.10%	1
CD22 Mutation	0.00%	0.10%	-0.10%	1
IGF1 Mutation	0.00%	0.10%	-0.10%	1
SRSF2 Mutation	0.00%	0.10%	-0.10%	1
CD70 Mutation	0.00%	0.10%	-0.10%	1
ETS1 Mutation	0.00%	0.10%	-0.10%	1
GATA3 Mutation	0.00%	0.10%	-0.10%	1
CD79A Mutation	0.00%	0.10%	-0.10%	1
MEF2C Mutation	0.00%	0.10%	-0.10%	1
SF3B1 Mutation	0.00%	0.10%	-0.10%	1
IGL Mutation	0.31%	0.20%	0.11%	1
CDK12 Mutation	0.31%	0.40%	-0.09%	1
HIST1H3B Mutation	0.00%	0.10%	-0.10%	1
TCL1A Mutation	0.00%	0.10%	-0.10%	1
PBX1 Mutation	0.00%	0.10%	-0.10%	1
TAF15 Mutation	0.00%	0.10%	-0.10%	1
TAL1 Mutation	0.00%	0.10%	-0.10%	1
CDKN1A Mutation	0.00%	0.10%	-0.10%	1
SSX1 Mutation	0.00%	0.10%	-0.10%	1
MEN1 CNA	0.31%	0.30%	0.01%	1
HGF CNA	0.31%	0.30%	0.01%	1
IGF1 CNA	0.31%	0.30%	0.01%	1
AKT3 CNA	0.31%	0.30%	0.01%	1
NBN Mutation	0.31%	0.30%	0.01%	1
BRIP1 Mutation	0.31%	0.30%	0.01%	1
MPL Mutation	0.31%	0.30%	0.01%	1
MSH6 Mutation	0.61%	0.70%	-0.08%	1

Supplementary Table 2: Genes Associated with Deficiencies in the Homologous Recombination Pathway

Supplementary Table 2: Genes Associated with Deficiencies in the Homologous Recombination Pathway			
Gene	Correlated to Increased gLOH	Correlated to PARP Inhibitor Monotherapy Sensitivity In Clinical Trials	Identified in LMS
ATM	25	40,41,53	8,43,44,47
ATR	25	53	45,46
BARD1	25		48
BRIP1	25		47
BRCA1	25	25,40,41,53	9,47,49
BRCA2	25	25,40,41,53	8-11,47
CHEK2	25	40,41,53	8,42
FANCA	25	40,53	8
FANCC	25	40	47
MRE11	25	53	47
NBN	25	53	
PALB2	25	41,53	9,50
RAD50	25		8,47,51
RAD51	25		8,52
RAD51B	25		6,11
RAD51C	25	19,53	
RAD51D	25	19	42
RAD52	25		
RAD54L	25		

Supplementary Table 3: Univariate Linear Regression of Genomic Loss of Heterozygosity and Alterations in the Homologous Recombination Pathway

Supplementary Table 3: Univariate Linear Regression of Genomic Loss of Heterozygosity and Alterations in the Homologous Recombination Pathway						
Gene	Alteration	n	Prevalence	Estimate (95%CI)	p Value	Adjusted p Value
BRCA2	Deletion	34	2.1%	6.13 (3.77 - 8.48)	3.729E-07	1.902E-05
BARD1	Truncation	1	0.1%	17.92 (4.23 - 31.6)	0.0103091	0.5257629
FANCA	Missense	4	0.2%	7.21 (0.36 - 14.06)	0.0392396	1
RAD51B	Rearrangement	2	0.1%	-9.21 (-18.9 - 0.48)	0.0623593	1
FANCA	Truncation	1	0.1%	11.93 (-1.75 - 25.62)	0.0874457	1
RAD50	Truncation	2	0.1%	-8.27 (-17.95 - 1.42)	0.0943061	1
BRCA2	Missense	10	0.6%	3.48 (-0.83 - 7.79)	0.1139183	1
MRE11	Missense	5	0.3%	-4.38 (-10.52 - 1.75)	0.1611538	1
NBN	Missense	4	0.2%	-4.72 (-11.58 - 2.13)	0.1769235	1
RAD51	Truncation	1	0.1%	-9.12 (-22.82 - 4.58)	0.1918804	1
BRCA2	Rearrangement	10	0.6%	-2.8 (-7.11 - 1.51)	0.2024581	1
BRCA1	Missense	3	0.2%	5.04 (-2.88 - 12.96)	0.2125367	1
RAD50	Missense	7	0.4%	-3.17 (-8.35 - 2.02)	0.2313382	1
MRE11	Truncation	2	0.1%	5.91 (-3.78 - 15.6)	0.2314412	1
RAD51B	Truncation	1	0.1%	-7.87 (-21.57 - 5.83)	0.2597787	1
NBN	Truncation	1	0.1%	-7.27 (-20.97 - 6.43)	0.2981203	1
RAD54L	Missense	5	0.3%	-3.12 (-9.26 - 3.01)	0.3183887	1
ATR	Missense	8	0.5%	2.41 (-2.45 - 7.27)	0.3303522	1
RAD51C	Missense	1	0.1%	6.74 (-6.97 - 20.44)	0.3350134	1
ATR	Rearrangement	3	0.2%	3.36 (-4.56 - 11.28)	0.4053855	1
BRIP1	Truncation	1	0.1%	-5.5 (-19.21 - 8.21)	0.4312444	1
CHEK2	Deletion	4	0.2%	2.72 (-4.14 - 9.58)	0.4369432	1
BRCA1	Truncation	1	0.1%	-5.36 (-19.07 - 8.35)	0.4435109	1
FANCC	Rearrangement	1	0.1%	-5.2 (-18.9 - 8.51)	0.4573857	1
CHEK2	Rearrangement	2	0.1%	-3.54 (-13.24 - 6.16)	0.4737053	1
PALB2	Rearrangement	1	0.1%	4.85 (-8.86 - 18.56)	0.4880397	1
ATM	Truncation	3	0.2%	-2.5 (-10.42 - 5.42)	0.5359772	1
BARD1	Deletion	2	0.1%	3 (-6.68 - 12.68)	0.5434354	1
MRE11	Rearrangement	2	0.1%	2.8 (-6.89 - 12.49)	0.5703996	1
BRIP1	Missense	3	0.2%	-2.28 (-10.2 - 5.64)	0.5724879	1
FANCA	Deletion	4	0.2%	1.9 (-4.95 - 8.75)	0.5868222	1
RAD51C	Rearrangement	1	0.1%	-3.61 (-17.32 - 10.09)	0.6053484	1
PALB2	Deletion	1	0.1%	-3.56 (-17.27 - 10.15)	0.6105203	1
BRCA2	Truncation	2	0.1%	2.48 (-7.13 - 12.1)	0.6123915	1
BRIP1	Rearrangement	1	0.1%	-3.44 (-17.15 - 10.27)	0.6223998	1
ATM	Missense	5	0.3%	-1.47 (-7.61 - 4.67)	0.6386185	1
RAD54L	Truncation	1	0.1%	3.28 (-10.43 - 16.98)	0.6389236	1

FANCA	Rearrangement	2	0.1%	-2.14 (-11.82 - 7.54)	0.6651506	1
BARD1	Rearrangement	1	0.1%	-2.88 (-16.57 - 10.8)	0.6797674	1
ATM	Deletion	1	0.1%	-2.73 (-16.45 - 10.98)	0.6962095	1
ATM	Rearrangement	4	0.2%	1.34 (-5.52 - 8.21)	0.7014457	1
FANCC	Missense	1	0.1%	2.31 (-11.39 - 16.02)	0.7405083	1
RAD51B	Deletion	38	2.3%	-0.24 (-2.49 - 2.01)	0.8339024	1
RAD51B	Missense	3	0.2%	0.65 (-7.26 - 8.57)	0.8714205	1
CHEK2	Truncation	1	0.1%	-1.1 (-14.82 - 12.61)	0.8745406	1
CHEK2	Missense	15	0.9%	-0.27 (-3.83 - 3.28)	0.8795302	1
BRCA1	Deletion	1	0.1%	1.04 (-12.67 - 14.75)	0.8814551	1
ATR	Truncation	3	0.2%	-0.47 (-8.39 - 7.45)	0.9067314	1
RAD51D	Missense	1	0.1%	0.81 (-12.9 - 14.51)	0.9080566	1
BRCA1	Rearrangement	2	0.1%	-0.56 (-10.26 - 9.13)	0.9094362	1
PALB2	Missense	7	0.4%	0.29 (-4.9 - 5.48)	0.9123083	1

Supplementary Table 4: Univariate Linear Regression of Genomic Loss of Heterozygosity and Genomic Alterations

Supplementary Table 4: Univariate Linear Regression of Genomic Loss of Heterozygosity and Genomic Alterations

Gene	Alteration	n	Prevalence	Estimate (95%CI)	p Value	Adjusted p Value
FBXW7	Amplification	8	0.005	18.75 (13.99 - 23.52)	2.03729E-14	1.47092E-11
NF1	Deletion	20	0.012	10.67 (7.65 - 13.69)	6.27602E-12	4.53128E-09
FHIT	Deletion	2	0.001	27.08 (17.47 - 36.69)	3.77207E-08	2.72344E-05
PTPN2	Amplification	6	0.004	15.03 (9.48 - 20.59)	1.25965E-07	9.09466E-05
BRCA2	Deletion	34	0.021	6.13 (3.77 - 8.48)	3.72857E-07	0.000269203
SRSF2	Missense	1	0.001	34.57 (20.96 - 48.17)	6.8958E-07	0.000497877
RB1	Deletion	515	0.311	1.9 (1.11 - 2.69)	2.30734E-06	0.001665897
CDKN2A	Deletion	118	0.071	3.13 (1.82 - 4.43)	2.66429E-06	0.001923621
CDKN2B	Deletion	99	0.06	3.11 (1.7 - 4.53)	1.63332E-05	0.011792538
SMC1A	Missense	1	0.001	29.94 (16.31 - 43.58)	1.73605E-05	0.012534263
MYC	Amplification	30	0.018	5.44 (2.93 - 7.95)	2.25994E-05	0.016316797
DAXX	Deletion	9	0.005	9.54 (4.99 - 14.08)	4.0136E-05	0.028978225
IKZF3	Deletion	1	0.001	28.18 (14.54 - 41.82)	5.28564E-05	0.038162322
BRAF	Amplification	4	0.002	13.12 (6.29 - 19.95)	0.000169745	0.122555706
NF1	Missense	15	0.009	6.61 (3.13 - 10.1)	0.000203945	0.147248065
KMT2D	Truncation	9	0.005	8.2 (3.63 - 12.76)	0.000436901	0.315442837
PIK3R1	Rearrangement	2	0.001	17.23 (7.58 - 26.88)	0.000473438	0.341821995
TBL1XR1	Amplification	4	0.002	12 (5.17 - 18.82)	0.000583825	0.421521321
ESR1	Amplification	15	0.009	6.22 (2.67 - 9.76)	0.000589965	0.425954456
CD22	Missense	1	0.001	23.51 (9.85 - 37.17)	0.000752609	0.543383761
TET2	Missense	11	0.007	6.69 (2.56 - 10.83)	0.001526944	1
LRP1B	Missense	16	0.01	5.46 (2.03 - 8.9)	0.001826701	1
TSC1	Deletion	4	0.002	10.41 (3.57 - 17.25)	0.002892923	1

PTEN	Deletion	200	0.121	1.53 (0.5 - 2.57)	0.00367863	1
CDKN1B	Deletion	8	0.005	6.99 (2.15 - 11.84)	0.004710623	1
NF1	Rearrangement	16	0.01	4.84 (1.46 - 8.21)	0.004975238	1
ALK	Amplification	1	0.001	19.46 (5.78 - 33.14)	0.005325905	1
PIK3CA	Amplification	5	0.003	8.69 (2.57 - 14.81)	0.005447598	1
KDM5A	Amplification	12	0.007	5.59 (1.63 - 9.55)	0.0057045	1
PASK	Truncation	4	0.002	9.58 (2.73 - 16.42)	0.006127115	1
CASP8	Deletion	1	0.001	19.06 (5.38 - 32.74)	0.006343152	1
BCOR	Rearrangement	6	0.004	-7.76 (-13.35 - -2.17)	0.006571618	1
CCND3	Amplification	14	0.008	5.08 (1.41 - 8.75)	0.006712602	1
KAT6A	Amplification	19	0.011	4.33 (1.17 - 7.48)	0.007235975	1
DNMT3A	Deletion	5	0.003	8.36 (2.24 - 14.49)	0.007492276	1
KRAS	Amplification	10	0.006	5.9 (1.57 - 10.23)	0.007624914	1
KRAS	Missense	5	0.003	8.32 (2.2 - 14.43)	0.007688461	1
DAXX	Truncation	5	0.003	8.26 (2.17 - 14.35)	0.007862921	1
PLCG2	Missense	2	0.001	12.86 (3.18 - 22.53)	0.009229293	1
BARD1	Truncation	1	0.001	17.92 (4.23 - 31.6)	0.010309077	1
NCOA2	Rearrangement	2	0.001	12.61 (2.94 - 22.29)	0.010659326	1
TP53	Rearrangement	42	0.025	2.87 (0.66 - 5.08)	0.01104238	1
SPEN	Rearrangement	1	0.001	17.56 (3.88 - 31.24)	0.011905409	1
HMGA2	Rearrangement	10	0.006	-5.49 (-9.83 - -1.15)	0.013182913	1
TCL1A	Missense	1	0.001	17.02 (3.34 - 30.7)	0.014812248	1
DAXX	Missense	22	0.013	3.56 (0.64 - 6.48)	0.016852136	1
NTRK1	Missense	1	0.001	16.43 (2.75 - 30.12)	0.018624339	1
TSC2	Missense	9	0.005	5.47 (0.9 - 10.04)	0.018970531	1
PIK3R1	Deletion	8	0.005	5.72 (0.88 - 10.55)	0.020439365	1
ATRX	Missense	184	0.111	1.28 (0.2 - 2.36)	0.020665382	1
FBXW7	Missense	4	0.002	-7.89 (-14.62 - -1.15)	0.021710591	1
ASXL1	Missense	5	0.003	-7.18 (-13.3 - -1.05)	0.021785502	1
RPTOR	Amplification	5	0.003	7.05 (0.92 - 13.18)	0.02416053	1
NSD1	Rearrangement	2	0.001	11.1 (1.42 - 20.79)	0.024631649	1

RB1	Rearrangement	97	0.059	1.68 (0.21 - 3.15)	0.025516385	1
CD274	Amplification	9	0.005	5.21 (0.63 - 9.78)	0.025656264	1
PDCD1LG2	Amplification	9	0.005	5.21 (0.63 - 9.78)	0.025733723	1
EGFR	Amplification	12	0.007	4.37 (0.41 - 8.34)	0.030579228	1
NCOR2	Missense	9	0.005	5.02 (0.45 - 9.6)	0.031460272	1
MSH6	Rearrangement	2	0.001	-10.62 (-20.31 - -0.94)	0.031578746	1
ZMYM3	Missense	3	0.002	-8.63 (-16.53 - -0.72)	0.032409794	1
PDGFRA	Amplification	18	0.011	3.53 (0.29 - 6.77)	0.032859617	1
FLCN	Rearrangement	3	0.002	-8.59 (-16.5 - -0.68)	0.033272003	1
MEN1	Missense	5	0.003	6.65 (0.51 - 12.78)	0.033656879	1
PICALM	Rearrangement	4	0.002	7.28 (0.43 - 14.13)	0.037167797	1
FANCA	Missense	4	0.002	7.21 (0.36 - 14.06)	0.039239635	1
FGF19	Amplification	7	0.004	5.45 (0.27 - 10.63)	0.039344881	1
FGF3	Amplification	7	0.004	5.45 (0.27 - 10.63)	0.039344881	1
FGF4	Amplification	7	0.004	5.45 (0.27 - 10.63)	0.039344881	1
CIC	Deletion	24	0.014	-2.93 (-5.75 - -0.12)	0.041188118	1
FGFR1	Amplification	24	0.014	2.91 (0.1 - 5.73)	0.042492462	1
CD58	Deletion	2	0.001	9.98 (0.29 - 19.66)	0.043432225	1
KIT	Amplification	16	0.01	3.53 (0.1 - 6.97)	0.043913641	1
TAF1	Missense	3	0.002	8.09 (0.18 - 16)	0.045144161	1
MAF	Amplification	3	0.002	8.08 (0.16 - 15.99)	0.045466372	1
APC	Rearrangement	4	0.002	7 (0.14 - 13.85)	0.045494138	1
JUN	Amplification	21	0.013	3.05 (0.04 - 6.06)	0.04670987	1
SDHB	Rearrangement	1	0.001	13.87 (0.18 - 27.57)	0.047103458	1
SUZ12	Rearrangement	1	0.001	13.82 (0.13 - 27.52)	0.047940964	1
FBXO11	Rearrangement	1	0.001	13.8 (0.12 - 27.49)	0.048100895	1
TP53	Deletion	253	0.153	1.09 (0.01 - 2.17)	0.048818435	1
FOXO1	Rearrangement	6	0.004	5.62 (0.02 - 11.21)	0.049210457	1
FGFR3	Amplification	6	0.004	5.61 (0.02 - 11.21)	0.049306266	1
TSC2	Truncation	2	0.001	9.7 (0.03 - 19.38)	0.049374898	1

SUFU	Missense	4	0.002	6.74 (-0.1 - 13.59)	0.05358459	1
PMS2	Missense	4	0.002	-6.72 (-13.58 - 0.13)	0.054485977	1
SUFU	Deletion	9	0.005	4.48 (-0.09 - 9.06)	0.054620041	1
ATRX	Deletion	98	0.059	1.4 (-0.03 - 2.84)	0.055287347	1
RAD51B	Rearrangement	2	0.001	-9.21 (-18.9 - 0.48)	0.062359294	1
HDAC4	Deletion	4	0.002	6.5 (-0.36 - 13.36)	0.063114682	1
TBL1XR1	Rearrangement	3	0.002	7.46 (-0.43 - 15.34)	0.063703557	1
DDX3X	Missense	1	0.001	-12.95 (-26.64 - 0.74)	0.063741655	1
RASGEF1A	Truncation	1	0.001	-12.95 (-26.65 - 0.74)	0.063743268	1
SMARCB1	Missense	1	0.001	-12.95 (-26.64 - 0.74)	0.063815079	1
JARID2	Missense	1	0.001	-12.95 (-26.65 - 0.75)	0.063847074	1
FGFR2	Missense	1	0.001	-12.95 (-26.64 - 0.75)	0.063874779	1
NUP98	Missense	1	0.001	-12.94 (-26.62 - 0.75)	0.063889517	1
CCND1	Missense	1	0.001	-12.94 (-26.63 - 0.75)	0.063962089	1
PBRM1	Truncation	3	0.002	-7.47 (-15.38 - 0.44)	0.064294609	1
CPS1	Missense	3	0.002	7.32 (-0.59 - 15.23)	0.069532019	1
PTCH1	Truncation	1	0.001	12.59 (-1.11 - 26.28)	0.071556684	1
CCNE1	Amplification	37	0.022	2.09 (-0.19 - 4.37)	0.071816486	1
TBX3	Missense	1	0.001	-12.56 (-26.25 - 1.13)	0.072166863	1
NRAS	Missense	1	0.001	-12.56 (-26.25 - 1.14)	0.072294961	1
INPP4B	Missense	1	0.001	-12.52 (-26.22 - 1.18)	0.073202468	1
ROS1	Missense	1	0.001	-12.51 (-26.21 - 1.18)	0.073289633	1
FOXL2	Missense	1	0.001	-12.49 (-26.18 - 1.2)	0.073771506	1

ARHGAP26	Missense	2	0.001	-8.81 (-18.5 - 0.87)	0.074463255	1
ZMYM3	Rearrangement	2	0.001	8.79 (-0.89 - 18.47)	0.074940467	1
FLT1	Rearrangement	1	0.001	12.31 (-1.38 - 26.01)	0.077949843	1
PBX1	Rearrangement	1	0.001	12.3 (-1.39 - 26)	0.078192073	1
IKBKE	Amplification	2	0.001	8.64 (-1.05 - 18.32)	0.080474829	1
ABL1	Missense	2	0.001	-8.61 (-18.3 - 1.08)	0.081448961	1
NBEAP1	Rearrangement	1	0.001	12.09 (-1.6 - 25.79)	0.083421858	1
ARAF	Amplification	6	0.004	4.94 (-0.66 - 10.54)	0.08385507	1
MYCL	Amplification	3	0.002	6.96 (-0.95 - 14.87)	0.084640576	1
CD79A	Missense	1	0.001	-11.98 (-25.67 - 1.71)	0.086361272	1
FANCA	Truncation	1	0.001	11.93 (-1.75 - 25.62)	0.087445672	1
MET	Amplification	6	0.004	4.87 (-0.73 - 10.47)	0.0882345	1
FAS	Deletion	36	0.022	2 (-0.31 - 4.31)	0.089627781	1
GAS7	Rearrangement	1	0.001	11.82 (-1.87 - 25.52)	0.090557928	1
CDC73	Missense	1	0.001	-11.8 (-25.5 - 1.9)	0.09127882	1
FAF1	Rearrangement	3	0.002	-6.82 (-14.73 - 1.1)	0.091359324	1
FGF6	Amplification	11	0.007	3.56 (-0.58 - 7.71)	0.091679566	1
RAD50	Truncation	2	0.001	-8.27 (-17.95 - 1.42)	0.094306111	1
NUP98	Rearrangement	4	0.002	5.74 (-1.11 - 12.59)	0.100235653	1
NF2	Deletion	3	0.002	6.57 (-1.34 - 14.48)	0.103464372	1
HGF	Amplification	5	0.003	5.08 (-1.06 - 11.21)	0.104691969	1
NF2	Rearrangement	2	0.001	-8 (-17.68 - 1.69)	0.10554202	1
FGF10	Amplification	30	0.018	2.08 (-0.44 - 4.6)	0.105678198	1
AURKA	Amplification	1	0.001	11.17 (-2.52 - 24.87)	0.109756007	1
NOTCH3	Deletion	1	0.001	11.17 (-2.53 - 24.86)	0.109966545	1
CREBBP	Truncation	3	0.002	-6.45 (-14.36 - 1.47)	0.110324238	1
BRCA2	Missense	10	0.006	3.48 (-0.83 - 7.79)	0.113918266	1
FANCE	Truncation	1	0.001	11.02 (-2.68 - 24.71)	0.114734535	1
RUNX1T1	Missense	2	0.001	-7.75 (-17.44 - 1.94)	0.116929901	1

CD70	Missense	1	0.001	-10.84 (-24.54 - 2.86)	0.1207801	1
PASK	Missense	12	0.007	3.1 (-0.86 - 7.06)	0.124518652	1
GATA1	Missense	1	0.001	-10.73 (-24.43 - 2.97)	0.124601559	1
AR	Amplification	7	0.004	4.05 (-1.14 - 9.23)	0.126214488	1
TOP1	Amplification	10	0.006	3.37 (-0.97 - 7.71)	0.128294502	1
LRP1B	Deletion	4	0.002	5.26 (-1.58 - 12.1)	0.131881056	1
MSH2	Deletion	3	0.002	6.04 (-1.88 - 13.96)	0.135075777	1
FANCM	Truncation	1	0.001	10.31 (-3.39 - 24.01)	0.14010213	1
HDAC1	Missense	1	0.001	10.27 (-3.42 - 23.97)	0.141479128	1
IDH1	Truncation	1	0.001	10.27 (-3.42 - 23.97)	0.141479128	1
FHIT	Rearrangement	1	0.001	-10.11 (-23.69 - 3.48)	0.144733829	1
PDGFRB	Missense	7	0.004	-3.86 (-9.04 - 1.33)	0.144986516	1
CTNNA1	Missense	2	0.001	7.19 (-2.51 - 16.88)	0.146057734	1
CPS1	Truncation	2	0.001	7.17 (-2.51 - 16.86)	0.146526398	1
FANCL	Missense	1	0.001	-10.13 (-23.83 - 3.57)	0.147276549	1
TLL2	Rearrangement	1	0.001	10.1 (-3.6 - 23.8)	0.148529689	1
MSH3	Truncation	1	0.001	10.07 (-3.63 - 23.77)	0.149524744	1
ECT2L	Missense	1	0.001	-10 (-23.7 - 3.7)	0.152393494	1
MKI67	Missense	4	0.002	-5 (-11.85 - 1.86)	0.15311996	1
NF1	Truncation	12	0.007	2.82 (-1.07 - 6.71)	0.155379034	1
PTPN2	Missense	1	0.001	9.84 (-3.75 - 23.42)	0.155791101	1
EGFR	Missense	1	0.001	-9.9 (-23.58 - 3.79)	0.156150509	1
BRAF	Missense	5	0.003	-4.4 (-10.51 - 1.71)	0.158325829	1
STAG2	Truncation	3	0.002	5.7 (-2.22 - 13.62)	0.158504682	1
NTRK3	Rearrangement	2	0.001	6.95 (-2.74 - 16.64)	0.159636621	1
PTEN	Missense	59	0.036	1.3 (-0.51 - 3.12)	0.1599138	1
MRE11	Missense	5	0.003	-4.38 (-10.52 - 1.75)	0.161153808	1
PDGFB	Rearrangement	2	0.001	-6.92 (-16.61 - 2.77)	0.161351685	1

CCND2	Amplification	12	0.007	2.83 (-1.14 - 6.8)	0.161667216	1
FGF23	Amplification	12	0.007	2.83 (-1.14 - 6.8)	0.162075522	1
PCLO	Missense	4	0.002	-4.86 (-11.72 - 2)	0.16456738	1
PDCD11	Missense	1	0.001	-9.71 (-23.41 - 3.99)	0.164658571	1
PTCH1	Missense	5	0.003	-4.34 (-10.47 - 1.79)	0.164727111	1
RAD21	Amplification	25	0.015	1.95 (-0.81 - 4.72)	0.165113658	1
MTOR	Amplification	2	0.001	6.76 (-2.93 - 16.46)	0.171158716	1
KMT2A	Rearrangement	1	0.001	-9.55 (-23.25 - 4.15)	0.171735977	1
PRDM1	Missense	6	0.004	3.9 (-1.7 - 9.51)	0.171824097	1
BRSK1	Missense	1	0.001	9.55 (-4.15 - 23.24)	0.17191259	1
NTRK2	Rearrangement	1	0.001	-9.54 (-23.24 - 4.16)	0.172185753	1
FLT3	Amplification	9	0.005	3.17 (-1.41 - 7.75)	0.17468127	1
EP300	Truncation	1	0.001	-9.47 (-23.18 - 4.24)	0.175614839	1
CDKN2C	Missense	1	0.001	-9.45 (-23.15 - 4.26)	0.176452459	1
NBN	Missense	4	0.002	-4.72 (-11.58 - 2.13)	0.176923498	1
NOTCH3	Truncation	2	0.001	6.67 (-3.02 - 16.36)	0.176935514	1
RUNX1	Deletion	1	0.001	9.43 (-4.28 - 23.14)	0.177340246	1
MAP2K2	Missense	1	0.001	-9.41 (-23.12 - 4.29)	0.177935307	1
SDHA	Truncation	3	0.002	5.44 (-2.48 - 13.36)	0.177952146	1
DUSP9	Missense	1	0.001	-9.41 (-23.11 - 4.29)	0.178113197	1
BCL2L2	Amplification	13	0.008	2.62 (-1.2 - 6.43)	0.178551565	1
RNF213	Rearrangement	2	0.001	-6.62 (-16.31 - 3.07)	0.180662835	1
MLH1	Deletion	3	0.002	5.32 (-2.6 - 13.23)	0.187701931	1
TBL1XR1	Missense	1	0.001	-9.17 (-22.81 - 4.48)	0.187803106	1
FBXO11	Deletion	2	0.001	6.48 (-3.2 - 16.16)	0.189233023	1
FAT3	Missense	3	0.002	5.3 (-2.62 - 13.21)	0.189498484	1
CCND1	Amplification	6	0.004	3.74 (-1.86 - 9.34)	0.190080465	1
IKZF3	Rearrangement	1	0.001	9.1 (-4.54 - 22.74)	0.190780953	1
MSH6	Deletion	2	0.001	6.45 (-3.23 - 16.14)	0.191370064	1
LEF1	Missense	1	0.001	-9.13 (-22.83 - 4.57)	0.191393027	1
RAD51	Truncation	1	0.001	-9.12 (-22.82 - 4.58)	0.191880417	1

CREBBP	Deletion	1	0.001	9.07 (-4.63 - 22.77)	0.194132255	1
FGF12	Amplification	6	0.004	3.68 (-1.92 - 9.28)	0.197710217	1
NOTCH3	Rearrangement	5	0.003	-4 (-10.14 - 2.13)	0.200594392	1
BCL7A	Rearrangement	3	0.002	5.17 (-2.75 - 13.08)	0.200621006	1
KMT2C	Deletion	1	0.001	8.91 (-4.79 - 22.62)	0.202312796	1
BRCA2	Rearrangement	10	0.006	-2.8 (-7.11 - 1.51)	0.202458115	1
CD36	Missense	27	0.016	1.72 (-0.93 - 4.38)	0.203474185	1
CIC	Truncation	2	0.001	-6.26 (-15.94 - 3.43)	0.205290459	1
NFKBIA	Missense	1	0.001	-8.83 (-22.53 - 4.87)	0.206351115	1
MUTYH	Missense	37	0.022	1.45 (-0.83 - 3.73)	0.211471299	1
EZH2	Missense	1	0.001	8.72 (-4.99 - 22.43)	0.212244038	1
BRCA1	Missense	3	0.002	5.04 (-2.88 - 12.96)	0.212536705	1
GATA2	Rearrangement	1	0.001	-8.68 (-22.38 - 5.02)	0.214237353	1
EED	Deletion	6	0.004	3.54 (-2.06 - 9.14)	0.215383381	1
SPEN	Missense	2	0.001	6.09 (-3.59 - 15.76)	0.21726956	1
MDM2	Amplification	33	0.02	-1.51 (-3.92 - 0.89)	0.217806334	1
EPHA3	Amplification	11	0.007	2.58 (-1.57 - 6.72)	0.222890871	1
PTPRO	Missense	1	0.001	-8.52 (-22.23 - 5.19)	0.223166764	1
PHF6	Deletion	1	0.001	-8.49 (-22.2 - 5.22)	0.224518008	1
ARID2	Rearrangement	1	0.001	-8.48 (-22.18 - 5.22)	0.224935646	1
CKS1B	Amplification	36	0.022	1.41 (-0.9 - 3.72)	0.230852602	1
XRCC3	Missense	2	0.001	5.92 (-3.77 - 15.61)	0.231099189	1
RAD50	Missense	7	0.004	-3.17 (-8.35 - 2.02)	0.231338189	1
MRE11	Truncation	2	0.001	5.91 (-3.78 - 15.6)	0.231441202	1
NTRK1	Amplification	25	0.015	1.67 (-1.09 - 4.43)	0.235111813	1
FGF7	Amplification	3	0.002	4.79 (-3.12 - 12.71)	0.23519222	1
ZNF703	Amplification	25	0.015	1.67 (-1.09 - 4.43)	0.235550984	1
PC	Rearrangement	1	0.001	-8.29 (-21.99 - 5.41)	0.235572373	1
FANCE	Missense	1	0.001	8.28 (-5.42 - 21.97)	0.235926364	1
GATA3	Rearrangement	1	0.001	8.27 (-5.43 - 21.97)	0.236513265	1
KDM5A	Missense	1	0.001	8.25 (-5.42 - 21.93)	0.236686194	1

PLAG1	Rearrangement	3	0.002	4.77 (-3.15 - 12.68)	0.237484895	1
MET	Missense	2	0.001	-5.83 (-15.52 - 3.85)	0.237689335	1
JAK2	Amplification	8	0.005	2.9 (-1.95 - 7.76)	0.241157604	1
PDGFRB	Rearrangement	2	0.001	5.77 (-3.92 - 15.45)	0.243100893	1
DNMT3A	Missense	21	0.013	1.79 (-1.22 - 4.79)	0.243194668	1
TLL2	Truncation	1	0.001	8.12 (-5.58 - 21.82)	0.245426857	1
PIK3R2	Rearrangement	3	0.002	4.67 (-3.25 - 12.58)	0.247864406	1
STK11	Missense	3	0.002	-4.65 (-12.57 - 3.27)	0.2495685	1
SMARCB1	Rearrangement	1	0.001	8.03 (-5.66 - 21.72)	0.249926893	1
RAF1	Amplification	1	0.001	8.04 (-5.67 - 21.74)	0.250104564	1
FRS2	Amplification	30	0.018	-1.46 (-3.99 - 1.06)	0.255094812	1
RAD51B	Truncation	1	0.001	-7.87 (-21.57 - 5.83)	0.259778679	1
ZNF217	Amplification	3	0.002	4.54 (-3.37 - 12.46)	0.260228908	1
CBL	Missense	4	0.002	3.92 (-2.94 - 10.78)	0.262177604	1
TGFBR2	Rearrangement	1	0.001	7.83 (-5.87 - 21.53)	0.26240849	1
TP53	Missense	834	0.503	0.46 (-0.35 - 1.27)	0.26321884	1
KMT2D	Missense	25	0.015	1.57 (-1.18 - 4.32)	0.263969497	1
CRKL	Amplification	22	0.013	1.67 (-1.26 - 4.61)	0.263979554	1
KDR	Missense	1	0.001	7.8 (-5.9 - 21.51)	0.264270069	1
TNFRSF14	Deletion	1	0.001	-7.8 (-21.5 - 5.9)	0.264450498	1
HLA-A	Missense	1	0.001	-7.78 (-21.48 - 5.92)	0.265513074	1
MAGED1	Missense	2	0.001	5.5 (-4.19 - 15.19)	0.265522529	1
SMARCA4	Truncation	2	0.001	-5.51 (-15.2 - 4.19)	0.265751323	1
TAL1	Rearrangement	1	0.001	7.77 (-5.93 - 21.47)	0.266085725	1
SOX2	Amplification	3	0.002	4.48 (-3.43 - 12.4)	0.266944207	1
FGFR3	Missense	1	0.001	7.74 (-5.95 - 21.43)	0.267766092	1
CHUK	Missense	1	0.001	7.7 (-6 - 21.41)	0.270613151	1
CTNNB1	Truncation	2	0.001	5.43 (-4.27 - 15.13)	0.272135979	1
PTPN6	Rearrangement	2	0.001	-5.38 (-15.07 - 4.32)	0.276595455	1
ROS1	Amplification	2	0.001	5.38 (-4.31 - 15.06)	0.276640189	1
AKT3	Amplification	5	0.003	-3.37 (-9.51 - 2.77)	0.281567063	1

SSX1	Rearrangement	1	0.001	7.48 (-6.22 - 21.18)	0.284357489	1
TRIM24	Rearrangement	1	0.001	7.43 (-6.27 - 21.13)	0.287591556	1
PAX5	Missense	2	0.001	-5.25 (-14.94 - 4.45)	0.288522513	1
NFKBIA	Amplification	7	0.004	2.81 (-2.38 - 7.99)	0.288994058	1
ICK	Missense	3	0.002	-4.28 (-12.19 - 3.64)	0.289654474	1
FAF1	Deletion	49	0.03	-1.07 (-3.06 - 0.91)	0.290135331	1
BIRC3	Amplification	8	0.005	2.61 (-2.24 - 7.47)	0.291209339	1
RAD21	Rearrangement	2	0.001	5.18 (-4.51 - 14.87)	0.294813433	1
NBN	Truncation	1	0.001	-7.27 (-20.97 - 6.43)	0.298120297	1
TSC1	Truncation	11	0.007	2.18 (-1.96 - 6.31)	0.301881001	1
ARID1A	Truncation	3	0.002	-4.17 (-12.09 - 3.75)	0.302319974	1
MPL	Truncation	1	0.001	7.19 (-6.51 - 20.9)	0.303517701	1
HNF1A	Missense	3	0.002	4.15 (-3.77 - 12.06)	0.304242292	1
BRD4	Missense	10	0.006	2.27 (-2.08 - 6.62)	0.306116325	1
KMT2D	Rearrangement	6	0.004	2.91 (-2.67 - 8.49)	0.306727738	1
SETD2	Missense	6	0.004	-2.91 (-8.51 - 2.7)	0.309450715	1
PARP4	Missense	2	0.001	-5.02 (-14.71 - 4.67)	0.309875361	1
IGF1R	Amplification	34	0.021	1.23 (-1.15 - 3.6)	0.31050466	1
BRSK1	Rearrangement	3	0.002	-4.08 (-12 - 3.83)	0.311474025	1
DAXX	Rearrangement	1	0.001	7.01 (-6.58 - 20.61)	0.311645613	1
CIITA	Missense	9	0.005	2.34 (-2.24 - 6.92)	0.316029379	1
ABL1	Rearrangement	1	0.001	6.97 (-6.72 - 20.66)	0.318234939	1
RAD54L	Missense	5	0.003	-3.12 (-9.26 - 3.01)	0.318388672	1
PBRM1	Missense	3	0.002	4 (-3.91 - 11.91)	0.321361305	1
CARD11	Missense	1	0.001	-6.89 (-20.59 - 6.82)	0.324334906	1
RB1	Missense	249	0.15	0.5 (-0.5 - 1.5)	0.32479714	1
HDAC4	Rearrangement	2	0.001	4.86 (-4.83 - 14.55)	0.32562423	1
ATR	Missense	8	0.005	2.41 (-2.45 - 7.27)	0.330352187	1
ERBB4	Amplification	1	0.001	6.79 (-6.91 - 20.5)	0.331211732	1
IKZF1	Rearrangement	1	0.001	6.79 (-6.92 - 20.5)	0.331419229	1
CD58	Missense	1	0.001	6.75 (-6.94 - 20.44)	0.333438002	1

CDKN2C	Deletion	53	0.032	-0.94 (-2.86 - 0.97)	0.333561879	1
AXL	Rearrangement	1	0.001	6.76 (-6.95 - 20.46)	0.333566048	1
BCL2	Amplification	9	0.005	2.26 (-2.32 - 6.84)	0.33381526	1
NOTCH2	Truncation	1	0.001	-6.75 (-20.46 - 6.95)	0.333987472	1
RAD51C	Missense	1	0.001	6.74 (-6.97 - 20.44)	0.335013365	1
IGF1	Missense	1	0.001	-6.72 (-20.43 - 6.98)	0.336215121	1
HIST1H3B	Missense	1	0.001	-6.72 (-20.42 - 6.99)	0.336431276	1
ETV6	Truncation	1	0.001	-6.72 (-20.43 - 6.99)	0.336640844	1
PARP1	Amplification	1	0.001	-6.69 (-20.39 - 7.02)	0.338595653	1
HIST1H1D	Missense	1	0.001	-6.69 (-20.39 - 7.02)	0.338595653	1
SMO	Missense	2	0.001	4.73 (-4.96 - 14.42)	0.338870769	1
SMAD4	Deletion	1	0.001	-6.68 (-20.39 - 7.02)	0.338879677	1
SUZ12	Truncation	1	0.001	6.67 (-7.02 - 20.37)	0.339444816	1
ESR1	Missense	6	0.004	-2.72 (-8.3 - 2.86)	0.339497714	1
POT1	Missense	2	0.001	4.69 (-5.01 - 14.38)	0.342987697	1
FOXP1	Rearrangement	4	0.002	-3.31 (-10.17 - 3.55)	0.344358203	1
TNFAIP3	Missense	2	0.001	4.67 (-5.02 - 14.36)	0.344528532	1
NCOR1	Deletion	8	0.005	-2.33 (-7.19 - 2.52)	0.345989724	1
MALT1	Missense	1	0.001	-6.59 (-20.3 - 7.12)	0.346053313	1
PAX5	Rearrangement	2	0.001	4.65 (-5.05 - 14.34)	0.347232779	1
SPEN	Truncation	1	0.001	6.53 (-7.15 - 20.21)	0.349342258	1
TSC1	Missense	2	0.001	4.61 (-5.06 - 14.29)	0.34959408	1
KMT2C	Truncation	9	0.005	-2.18 (-6.76 - 2.4)	0.351240721	1
SMAD2	Missense	1	0.001	6.51 (-7.19 - 20.21)	0.351517847	1
U2AF1	Missense	1	0.001	-6.51 (-20.21 - 7.2)	0.351768872	1
FGF14	Missense	1	0.001	-6.49 (-20.2 - 7.22)	0.353131865	1
PAK5	Missense	2	0.001	-4.58 (-14.28 - 5.11)	0.35374639	1
PRKDC	Rearrangement	1	0.001	6.42 (-7.28 - 20.12)	0.358221429	1
JAK1	Missense	1	0.001	-6.4 (-20.11 - 7.31)	0.359904397	1
ASXL1	Rearrangement	1	0.001	6.38 (-7.31 - 20.07)	0.360570729	1
CDC73	Truncation	1	0.001	-6.34 (-20.04 - 7.36)	0.364059593	1

FANCM	Missense	1	0.001	-6.33 (-20.03 - 7.37)	0.364804371	1
CPS1	Deletion	2	0.001	4.45 (-5.23 - 14.14)	0.367336704	1
MSH3	Missense	4	0.002	3.14 (-3.71 - 10)	0.368688839	1
FOXP1	Missense	3	0.002	-3.63 (-11.55 - 4.29)	0.368873875	1
MCL1	Amplification	36	0.022	1.06 (-1.25 - 3.36)	0.369827852	1
PRDM1	Rearrangement	1	0.001	-6.25 (-19.95 - 7.45)	0.370785755	1
TMEM30A	Missense	2	0.001	-4.39 (-14.09 - 5.3)	0.374082268	1
TYK2	Missense	2	0.001	-4.36 (-14.05 - 5.33)	0.37790567	1
MEF2C	Amplification	2	0.001	-4.35 (-14.04 - 5.34)	0.378877752	1
POT1	Truncation	1	0.001	-6.15 (-19.85 - 7.56)	0.379163757	1
BCORL1	Rearrangement	2	0.001	-4.34 (-14.04 - 5.36)	0.380089564	1
LRRK2	Missense	2	0.001	4.31 (-5.39 - 14)	0.383587291	1
AR	Missense	1	0.001	-6.02 (-19.72 - 7.68)	0.388822873	1
FGFR3	Rearrangement	1	0.001	-6.01 (-19.7 - 7.68)	0.389195793	1
ERBB3	Amplification	6	0.004	-2.46 (-8.06 - 3.15)	0.389689554	1
BAP1	Missense	2	0.001	-4.24 (-13.93 - 5.46)	0.391642953	1
ACTB	Rearrangement	2	0.001	-4.23 (-13.92 - 5.46)	0.39231628	1
GID4	Amplification	191	0.115	-0.46 (-1.51 - 0.59)	0.39273945	1
SH2B3	Rearrangement	1	0.001	5.95 (-7.76 - 19.66)	0.394694424	1
TUSC3	Missense	1	0.001	-5.95 (-19.65 - 7.76)	0.394790014	1
CUX1	Rearrangement	5	0.003	2.66 (-3.48 - 8.8)	0.395661015	1
IRS2	Amplification	23	0.014	1.24 (-1.63 - 4.12)	0.396449732	1
HRAS	Amplification	2	0.001	4.17 (-5.52 - 13.86)	0.398694275	1
IGF2	Amplification	2	0.001	4.17 (-5.52 - 13.86)	0.398694275	1
CDKN2A	Rearrangement	3	0.002	3.36 (-4.51 - 11.23)	0.402330447	1
NFKBIA	Truncation	1	0.001	-5.85 (-19.55 - 7.85)	0.402410999	1
MEN1	Truncation	4	0.002	2.92 (-3.93 - 9.77)	0.403025524	1
ATR	Rearrangement	3	0.002	3.36 (-4.56 - 11.28)	0.40538549	1
AKT1	Amplification	2	0.001	-4.11 (-13.81 - 5.59)	0.405784153	1
KDM5C	Missense	2	0.001	4.1 (-5.59 - 13.79)	0.406666481	1
TNFRSF11A	Amplification	6	0.004	2.37 (-3.23 - 7.97)	0.406800547	1

NF2	Truncation	3	0.002	3.33 (-4.58 - 11.24)	0.40899282	1
KIT	Missense	1	0.001	5.74 (-7.95 - 19.44)	0.410667806	1
HLA-A	Truncation	1	0.001	-5.74 (-19.44 - 7.96)	0.411293203	1
FBXO31	Rearrangement	1	0.001	5.73 (-7.98 - 19.44)	0.412252389	1
CTCF	Rearrangement	1	0.001	5.72 (-7.99 - 19.43)	0.41327752	1
DNMT3A	Truncation	5	0.003	-2.55 (-8.68 - 3.57)	0.413726054	1
FLCN	Missense	2	0.001	-4.03 (-13.71 - 5.66)	0.414802228	1
ICK	Rearrangement	3	0.002	3.28 (-4.63 - 11.2)	0.416303213	1
KAT6A	Rearrangement	2	0.001	4.01 (-5.67 - 13.68)	0.41681747	1
MEF2C	Rearrangement	1	0.001	5.65 (-8.05 - 19.36)	0.418451322	1
AKT3	Missense	3	0.002	3.26 (-4.66 - 11.18)	0.419497126	1
RICTOR	Amplification	44	0.027	0.86 (-1.23 - 2.95)	0.419720175	1
PHF6	Rearrangement	1	0.001	-5.56 (-19.27 - 8.15)	0.426292677	1
WT1	Truncation	1	0.001	-5.55 (-19.26 - 8.16)	0.427593232	1
NCOR1	Truncation	1	0.001	-5.54 (-19.25 - 8.17)	0.427950636	1
BRIP1	Truncation	1	0.001	-5.5 (-19.21 - 8.21)	0.431244448	1
FGFR4	Amplification	3	0.002	3.16 (-4.76 - 11.07)	0.434595497	1
PRSS8	Amplification	1	0.001	-5.45 (-19.15 - 8.26)	0.435768248	1
CHEK2	Deletion	4	0.002	2.72 (-4.14 - 9.58)	0.436943176	1
PTCH1	Rearrangement	2	0.001	3.84 (-5.85 - 13.52)	0.437317408	1
INPP5D	Missense	1	0.001	-5.43 (-19.13 - 8.28)	0.437456794	1
CBFB	Deletion	5	0.003	-2.43 (-8.57 - 3.71)	0.43811936	1
CDKN1B	Rearrangement	1	0.001	5.39 (-8.29 - 19.06)	0.440014988	1
ZMYM3	Truncation	3	0.002	-3.11 (-11.01 - 4.8)	0.440719415	1
EPHA7	Missense	1	0.001	-5.38 (-19.08 - 8.33)	0.441694581	1
MAP3K1	Rearrangement	1	0.001	-5.38 (-19.08 - 8.33)	0.441907963	1
PIK3R1	Truncation	1	0.001	5.35 (-8.3 - 18.99)	0.442138789	1
FLT3	Missense	1	0.001	5.37 (-8.33 - 19.07)	0.442379569	1
TSC2	Deletion	2	0.001	-3.78 (-13.46 - 5.89)	0.443075396	1
PRKAR1A	Truncation	2	0.001	3.79 (-5.91 - 13.49)	0.443341312	1
BRCA1	Truncation	1	0.001	-5.36 (-19.07 - 8.35)	0.44351086	1

WDR90	Missense	3	0.002	-3.09 (-11.01 - 4.82)	0.443725193	1
PDGFRA	Missense	4	0.002	-2.67 (-9.52 - 4.18)	0.444064628	1
NPM1	Rearrangement	1	0.001	-5.34 (-19.04 - 8.37)	0.445101653	1
POT1	Rearrangement	2	0.001	3.77 (-5.93 - 13.46)	0.445924518	1
TP63	Deletion	1	0.001	-5.32 (-19.04 - 8.39)	0.446376464	1
SDHC	Rearrangement	1	0.001	5.32 (-8.39 - 19.03)	0.446694437	1
IDH2	Missense	1	0.001	5.3 (-8.4 - 19)	0.448233088	1
IGL	Rearrangement	4	0.002	-2.65 (-9.51 - 4.21)	0.448257744	1
FAS	Rearrangement	1	0.001	5.24 (-8.46 - 18.94)	0.453013333	1
ARID2	Missense	6	0.004	2.14 (-3.46 - 7.74)	0.454329434	1
NCOR1	Rearrangement	10	0.006	1.65 (-2.7 - 6)	0.456692055	1
RPTOR	Missense	1	0.001	5.19 (-8.5 - 18.88)	0.457053022	1
FANCC	Rearrangement	1	0.001	-5.2 (-18.9 - 8.51)	0.457385664	1
MAP3K6	Truncation	6	0.004	2.12 (-3.48 - 7.73)	0.457544653	1
CCND3	Rearrangement	1	0.001	-5.17 (-18.85 - 8.5)	0.458267198	1
ALK	Missense	2	0.001	-3.65 (-13.33 - 6.02)	0.459057837	1
ASMTL	Missense	1	0.001	5.17 (-8.53 - 18.87)	0.459452965	1
RET	Rearrangement	2	0.001	3.64 (-6.06 - 13.34)	0.461834163	1
KDM6A	Missense	6	0.004	2.1 (-3.51 - 7.71)	0.462425946	1
SDHB	Truncation	1	0.001	5.05 (-8.64 - 18.75)	0.469413947	1
PIK3CA	Missense	21	0.013	-1.11 (-4.11 - 1.9)	0.46978005	1
CHEK2	Rearrangement	2	0.001	-3.54 (-13.24 - 6.16)	0.473705294	1
PIK3R2	Missense	1	0.001	-4.98 (-18.68 - 8.73)	0.476247996	1
GNAQ	Missense	1	0.001	-4.96 (-18.66 - 8.75)	0.478206047	1
DNMT3A	Rearrangement	4	0.002	-2.44 (-9.29 - 4.4)	0.484321155	1
BCL6	Missense	1	0.001	-4.88 (-18.59 - 8.82)	0.484751161	1
PALB2	Rearrangement	1	0.001	4.85 (-8.86 - 18.56)	0.488039672	1
TRAF2	Rearrangement	2	0.001	3.39 (-6.31 - 13.08)	0.493387964	1
EZH2	Truncation	1	0.001	-4.76 (-18.47 - 8.95)	0.495890927	1
SDHB	Deletion	2	0.001	3.35 (-6.33 - 13.04)	0.49737185	1
RB1	Truncation	83	0.05	-0.54 (-2.12 - 1.03)	0.498337653	1

DNM2	Missense	1	0.001	4.73 (-8.98 - 18.44)	0.49855685	1
RELN	Missense	6	0.004	-1.93 (-7.53 - 3.68)	0.50045487	1
PASK	Rearrangement	2	0.001	3.3 (-6.37 - 12.97)	0.503671242	1
MSH6	Truncation	2	0.001	-3.3 (-12.98 - 6.39)	0.504337785	1
MALT1	Truncation	1	0.001	-4.66 (-18.37 - 9.05)	0.505244601	1
STAT4	Deletion	1	0.001	4.65 (-9.05 - 18.36)	0.505647067	1
SMAD4	Truncation	1	0.001	4.65 (-9.06 - 18.35)	0.506247905	1
EPHA3	Missense	3	0.002	-2.67 (-10.59 - 5.24)	0.507650637	1
CDKN2A	Missense	15	0.009	-1.19 (-4.72 - 2.34)	0.509356671	1
CDK12	Rearrangement	2	0.001	3.25 (-6.45 - 12.95)	0.511228016	1
BCORL1	Deletion	2	0.001	-3.2 (-12.89 - 6.5)	0.518130661	1
AXL	Amplification	8	0.005	1.6 (-3.26 - 6.46)	0.518247001	1
FLT4	Amplification	1	0.001	4.49 (-9.22 - 18.2)	0.520676024	1
CDKN1A	Rearrangement	1	0.001	-4.47 (-18.17 - 9.24)	0.522806122	1
CUX1	Missense	16	0.01	-1.1 (-4.55 - 2.34)	0.529572051	1
MTOR	Missense	4	0.002	-2.18 (-9.03 - 4.68)	0.533850848	1
LRP1B	Rearrangement	4	0.002	2.17 (-4.67 - 9.01)	0.533925324	1
ATM	Truncation	3	0.002	-2.5 (-10.42 - 5.42)	0.535977205	1
CHD2	Missense	4	0.002	-2.15 (-9.01 - 4.71)	0.539029439	1
MAF	Rearrangement	1	0.001	4.29 (-9.41 - 17.98)	0.539499891	1
BARD1	Deletion	2	0.001	3 (-6.68 - 12.68)	0.543435382	1
PPP2R1A	Missense	1	0.001	-4.25 (-17.95 - 9.46)	0.543503137	1
PTPN11	Missense	1	0.001	-4.25 (-17.95 - 9.46)	0.543503137	1
REL	Amplification	2	0.001	3 (-6.69 - 12.69)	0.543888824	1
MLH1	Rearrangement	1	0.001	4.22 (-9.48 - 17.92)	0.545980884	1
MAP3K14	Amplification	4	0.002	-2.11 (-8.97 - 4.75)	0.546649673	1
PIM1	Amplification	8	0.005	1.49 (-3.37 - 6.35)	0.547311068	1
JAK3	Missense	1	0.001	4.14 (-9.57 - 17.84)	0.553677283	1
CDK8	Amplification	9	0.005	1.38 (-3.2 - 5.96)	0.553992438	1
CAD	Rearrangement	3	0.002	2.38 (-5.54 - 10.3)	0.555133662	1
RASGEF1A	Missense	1	0.001	-4.11 (-17.81 - 9.58)	0.555841225	1

MAP2K2	Amplification	3	0.002	2.37 (-5.55 - 10.28)	0.557885691	1
HDAC4	Truncation	1	0.001	-4.09 (-17.79 - 9.61)	0.558410348	1
HSP90AA1	Missense	2	0.001	-2.88 (-12.58 - 6.81)	0.560001164	1
NOTCH3	Amplification	11	0.007	-1.23 (-5.37 - 2.92)	0.561939997	1
GADD45B	Missense	1	0.001	-4.02 (-17.73 - 9.68)	0.5649078	1
NOTCH2	Rearrangement	3	0.002	-2.31 (-10.23 - 5.61)	0.566551844	1
CDKN1B	Truncation	1	0.001	3.99 (-9.69 - 17.66)	0.567672707	1
HGF	Truncation	2	0.001	-2.82 (-12.51 - 6.87)	0.567981211	1
BCR	Rearrangement	2	0.001	2.82 (-6.87 - 12.51)	0.568350531	1
MRE11	Rearrangement	2	0.001	2.8 (-6.89 - 12.49)	0.570399607	1
CPS1	Rearrangement	2	0.001	2.8 (-6.88 - 12.49)	0.570416832	1
BCL6	Rearrangement	1	0.001	3.97 (-9.74 - 17.67)	0.570437259	1
NKX2-1	Amplification	7	0.004	1.5 (-3.69 - 6.69)	0.571896799	1
STAT4	Missense	2	0.001	2.79 (-6.9 - 12.49)	0.572156172	1
BRIP1	Missense	3	0.002	-2.28 (-10.2 - 5.64)	0.572487864	1
PASK	Deletion	1	0.001	3.9 (-9.78 - 17.57)	0.576125834	1
BCL3	Rearrangement	1	0.001	3.89 (-9.82 - 17.59)	0.57789481	1
GADD45B	Deletion	1	0.001	3.89 (-9.82 - 17.59)	0.578209112	1
ATRX	Truncation	116	0.07	-0.38 (-1.7 - 0.95)	0.579461873	1
CIITA	Rearrangement	1	0.001	3.82 (-9.88 - 17.53)	0.584497326	1
EPHB1	Missense	1	0.001	-3.81 (-17.51 - 9.9)	0.586082102	1
FANCA	Deletion	4	0.002	1.9 (-4.95 - 8.75)	0.586822178	1
APC	Truncation	1	0.001	-3.79 (-17.48 - 9.91)	0.587656178	1
BCL10	Missense	2	0.001	2.67 (-7.02 - 12.36)	0.589128576	1
NSD1	Truncation	1	0.001	-3.76 (-17.45 - 9.93)	0.58990862	1
STAG2	Deletion	3	0.002	-2.17 (-10.09 - 5.75)	0.590951354	1
NCOR1	Missense	7	0.004	1.42 (-3.77 - 6.61)	0.591804386	1
RAF1	Rearrangement	2	0.001	-2.63 (-12.32 - 7.07)	0.595146362	1
CIC	Missense	6	0.004	-1.51 (-7.1 - 4.09)	0.597947182	1
RAD51C	Rearrangement	1	0.001	-3.61 (-17.32 - 10.09)	0.605348366	1

PALB2	Deletion	1	0.001	-3.56 (-17.27 - 10.15)	0.610520317	1
BRCA2	Truncation	2	0.001	2.48 (-7.13 - 12.1)	0.612391479	1
CTNNB1	Rearrangement	3	0.002	-2.04 (-9.96 - 5.88)	0.612979803	1
FGF23	Missense	1	0.001	-3.46 (-17.17 - 10.24)	0.619955302	1
HDAC4	Missense	3	0.002	-1.99 (-9.91 - 5.92)	0.621124579	1
BRIP1	Rearrangement	1	0.001	-3.44 (-17.15 - 10.27)	0.622399762	1
ASXL1	Truncation	3	0.002	1.98 (-5.93 - 9.89)	0.623567993	1
RUNX1	Rearrangement	4	0.002	-1.7 (-8.56 - 5.16)	0.626467286	1
BRAF	Rearrangement	4	0.002	-1.69 (-8.52 - 5.14)	0.627706287	1
SS18	Rearrangement	3	0.002	-1.96 (-9.87 - 5.96)	0.627912582	1
IGF1	Amplification	5	0.003	-1.51 (-7.65 - 4.62)	0.628546283	1
SF3B1	Missense	1	0.001	-3.37 (-17.07 - 10.34)	0.630145229	1
NOD1	Truncation	2	0.001	-2.36 (-12.06 - 7.34)	0.633358396	1
FANCD2	Missense	6	0.004	1.35 (-4.25 - 6.96)	0.636494414	1
CUL4A	Amplification	48	0.029	0.48 (-1.52 - 2.49)	0.636676444	1
CDKN2A	Truncation	3	0.002	-1.89 (-9.76 - 5.98)	0.637269978	1
CAD	Missense	1	0.001	3.29 (-10.42 - 17)	0.637585248	1
RET	Amplification	2	0.001	-2.33 (-12.02 - 7.37)	0.638160248	1
ATM	Missense	5	0.003	-1.47 (-7.61 - 4.67)	0.638618519	1
RAD54L	Truncation	1	0.001	3.28 (-10.43 - 16.98)	0.638923645	1
CBFB	Rearrangement	1	0.001	3.28 (-10.43 - 16.99)	0.638937328	1
BACH1	Deletion	1	0.001	3.28 (-10.44 - 17)	0.639305791	1
SOX10	Missense	1	0.001	-3.26 (-16.96 - 10.45)	0.641378163	1
TCF3	Rearrangement	4	0.002	-1.63 (-8.49 - 5.23)	0.642094884	1
NOTCH3	Missense	8	0.005	1.14 (-3.71 - 6)	0.643769463	1
LPP	Rearrangement	6	0.004	1.32 (-4.28 - 6.92)	0.644177449	1
NOTCH4	Deletion	1	0.001	-3.21 (-16.92 - 10.51)	0.646526854	1

RUNX1	Missense	1	0.001	3.18 (-10.53 - 16.89)	0.649082009	1
CASP8	Rearrangement	1	0.001	3.16 (-10.52 - 16.84)	0.650522552	1
STAG2	Rearrangement	1	0.001	3.16 (-10.55 - 16.86)	0.651632778	1
ATRX	Rearrangement	22	0.013	0.67 (-2.27 - 3.61)	0.653893105	1
TLL2	Missense	1	0.001	-3.12 (-16.82 - 10.58)	0.654721561	1
CDC73	Rearrangement	3	0.002	1.8 (-6.11 - 9.72)	0.655146214	1
FBXW7	Truncation	1	0.001	-3.06 (-16.52 - 10.39)	0.655153242	1
CREBBP	Rearrangement	9	0.005	1.03 (-3.55 - 5.61)	0.659051765	1
INPP4B	Truncation	1	0.001	-3.06 (-16.76 - 10.64)	0.661146836	1
TOP1	Missense	1	0.001	-3.03 (-16.74 - 10.67)	0.663998868	1
FANCG	Truncation	1	0.001	-3.04 (-16.75 - 10.67)	0.664156058	1
FANCA	Rearrangement	2	0.001	-2.14 (-11.82 - 7.54)	0.665150633	1
CSF1R	Truncation	1	0.001	3.02 (-10.69 - 16.72)	0.665817376	1
MAP3K6	Missense	16	0.01	0.75 (-2.69 - 4.2)	0.668096374	1
JAK1	Rearrangement	2	0.001	-2.11 (-11.8 - 7.59)	0.66956381	1
SUZ12	Missense	2	0.001	2.11 (-7.58 - 11.8)	0.669597643	1
NFE2L2	Missense	1	0.001	-2.98 (-16.68 - 10.73)	0.670338765	1
NRAS	Amplification	2	0.001	2.1 (-7.59 - 11.78)	0.671265613	1
MSH6	Missense	7	0.004	-1.12 (-6.3 - 4.07)	0.672210468	1
BACH1	Missense	1	0.001	2.93 (-10.79 - 16.65)	0.675483171	1
CD36	Truncation	5	0.003	-1.3 (-7.44 - 4.83)	0.67704583	1
SETD2	Rearrangement	1	0.001	-2.91 (-16.62 - 10.8)	0.677517022	1
SMARCA4	Deletion	1	0.001	-2.9 (-16.61 - 10.82)	0.678837089	1
BARD1	Rearrangement	1	0.001	-2.88 (-16.57 - 10.8)	0.67976738	1
IKZF1	Truncation	1	0.001	-2.88 (-16.59 - 10.83)	0.680189912	1

SDHA	Rearrangement	1	0.001	-2.85 (-16.57 - 10.86)	0.683078348	1
KEAP1	Rearrangement	2	0.001	-1.99 (-11.69 - 7.71)	0.687376477	1
KDM4C	Amplification	7	0.004	1.06 (-4.13 - 6.25)	0.689626416	1
PTEN	Truncation	21	0.013	-0.61 (-3.61 - 2.4)	0.692962202	1
ATM	Deletion	1	0.001	-2.73 (-16.45 - 10.98)	0.696209542	1
ATM	Rearrangement	4	0.002	1.34 (-5.52 - 8.21)	0.701445697	1
TRAF5	Missense	3	0.002	1.53 (-6.39 - 9.44)	0.70544451	1
INHBA	Missense	2	0.001	1.86 (-7.84 - 11.55)	0.706889158	1
NTRK1	Rearrangement	2	0.001	1.85 (-7.83 - 11.53)	0.70721648	1
MEN1	Deletion	5	0.003	1.17 (-4.96 - 7.3)	0.707916145	1
NOTCH4	Missense	6	0.004	-1.07 (-6.68 - 4.54)	0.708175671	1
PIK3C2G	Missense	5	0.003	-1.17 (-7.31 - 4.97)	0.708774357	1
SETD2	Truncation	1	0.001	-2.6 (-16.31 - 11.11)	0.710256554	1
FGF14	Amplification	18	0.011	0.61 (-2.63 - 3.86)	0.710591084	1
FGFR2	Amplification	4	0.002	1.29 (-5.57 - 8.14)	0.712705617	1
AURKB	Amplification	67	0.04	0.32 (-1.39 - 2.03)	0.71284603	1
NOD1	Rearrangement	3	0.002	1.49 (-6.44 - 9.41)	0.71305436	1
SUZ12	Deletion	1	0.001	2.54 (-11.15 - 16.24)	0.715787845	1
APCDD1	Missense	3	0.002	1.44 (-6.48 - 9.36)	0.721483816	1
CDK6	Amplification	2	0.001	1.76 (-7.94 - 11.45)	0.722002733	1
AXIN1	Missense	4	0.002	1.24 (-5.62 - 8.1)	0.722480533	1
CDK12	Truncation	1	0.001	-2.48 (-16.19 - 11.23)	0.722751053	1
MSH2	Rearrangement	2	0.001	1.75 (-7.95 - 11.44)	0.723867999	1
FBXO31	Deletion	1	0.001	2.45 (-11.26 - 16.16)	0.725778739	1
MAF	Missense	1	0.001	2.45 (-11.25 - 16.14)	0.726236898	1
MAP3K13	Missense	2	0.001	-1.73 (-11.42 - 7.97)	0.727073685	1
B2M	Missense	3	0.002	1.4 (-6.52 - 9.32)	0.728183327	1
SMARCD1	Missense	2	0.001	1.7 (-8 - 11.39)	0.731123641	1
BCORL1	Missense	5	0.003	-1.06 (-7.2 - 5.08)	0.735462384	1

EPHA5	Missense	3	0.002	-1.36 (-9.27 - 6.56)	0.737033127	1
CIC	Rearrangement	1	0.001	2.33 (-11.36 - 16.03)	0.738334018	1
JARID2	Truncation	2	0.001	-1.64 (-11.33 - 8.05)	0.73945727	1
FANCC	Missense	1	0.001	2.31 (-11.39 - 16.02)	0.740508278	1
TSC1	Rearrangement	2	0.001	1.62 (-8.05 - 11.29)	0.742744566	1
PTEN	Rearrangement	9	0.005	0.76 (-3.81 - 5.34)	0.742934834	1
BACH1	Rearrangement	3	0.002	-1.31 (-9.24 - 6.61)	0.745454275	1
BLM	Missense	2	0.001	1.6 (-8.09 - 11.3)	0.745694698	1
NF2	Missense	4	0.002	1.13 (-5.72 - 7.99)	0.745841441	1
SMARCA4	Rearrangement	10	0.006	-0.72 (-5.07 - 3.63)	0.745984282	1
MALT1	Amplification	3	0.002	1.3 (-6.62 - 9.22)	0.747095863	1
ALK	Rearrangement	30	0.018	0.41 (-2.11 - 2.93)	0.747389687	1
ARID1B	Rearrangement	7	0.004	-0.85 (-6.04 - 4.34)	0.748960901	1
PHF6	Missense	1	0.001	2.21 (-11.5 - 15.92)	0.751885964	1
PAX5	Deletion	1	0.001	2.21 (-11.5 - 15.91)	0.752137436	1
ERBB4	Missense	1	0.001	2.19 (-11.51 - 15.9)	0.753792466	1
TSC2	Rearrangement	4	0.002	-1.09 (-7.93 - 5.76)	0.755568061	1
TAF1	Rearrangement	1	0.001	2.17 (-11.52 - 15.87)	0.755691144	1
EMSY	Amplification	4	0.002	-1.07 (-7.93 - 5.79)	0.758931158	1
PDCD1LG2	Missense	1	0.001	-2.14 (-15.83 - 11.55)	0.759578751	1
AKT1	Missense	3	0.002	-1.23 (-9.15 - 6.69)	0.760572169	1
PBRM1	Deletion	3	0.002	-1.21 (-9.12 - 6.71)	0.765180795	1
BAP1	Deletion	3	0.002	-1.2 (-9.12 - 6.71)	0.765585108	1
TRAF3	Rearrangement	2	0.001	-1.46 (-11.15 - 8.23)	0.767727404	1
EP300	Missense	3	0.002	-1.18 (-9.1 - 6.74)	0.770346195	1
SUFU	Rearrangement	2	0.001	1.42 (-8.25 - 11.1)	0.772945111	1
PIK3CG	Amplification	2	0.001	-1.4 (-11.09 - 8.3)	0.777633169	1
FAF1	Truncation	2	0.001	-1.37 (-11.06 - 8.32)	0.780945096	1
MSH2	Missense	4	0.002	-0.97 (-7.83 - 5.89)	0.781986012	1
MDM4	Amplification	5	0.003	0.86 (-5.28 - 7)	0.783635501	1

CREBBP	Missense	6	0.004	-0.76 (-6.36 - 4.84)	0.789287438	1
FGFR1	Rearrangement	2	0.001	-1.3 (-10.99 - 8.38)	0.791963273	1
EP300	Rearrangement	5	0.003	0.81 (-5.33 - 6.95)	0.795448258	1
CDKN2C	Rearrangement	1	0.001	1.8 (-11.9 - 15.51)	0.796502673	1
CTNNB1	Missense	1	0.001	1.77 (-11.94 - 15.48)	0.800038182	1
GNAS	Amplification	5	0.003	-0.79 (-6.93 - 5.35)	0.800775545	1
B2M	Rearrangement	1	0.001	1.76 (-11.95 - 15.47)	0.801199413	1
PIK3CG	Missense	2	0.001	-1.23 (-10.93 - 8.47)	0.803331391	1
TP63	Missense	2	0.001	1.22 (-8.48 - 10.92)	0.805109051	1
ARID1A	Rearrangement	5	0.003	0.76 (-5.38 - 6.9)	0.808624623	1
FOXP1	Deletion	2	0.001	1.18 (-8.52 - 10.88)	0.810896002	1
TET2	Truncation	9	0.005	0.55 (-4.02 - 5.11)	0.814929646	1
CDK4	Amplification	35	0.021	0.28 (-2.06 - 2.62)	0.816610735	1
MAP3K1	Missense	1	0.001	1.61 (-12.09 - 15.32)	0.817344081	1
RET	Missense	1	0.001	1.61 (-12.1 - 15.32)	0.817964327	1
TCF3	Missense	2	0.001	-1.12 (-10.82 - 8.58)	0.821033621	1
CTCF	Truncation	1	0.001	-1.57 (-15.28 - 12.14)	0.822191881	1
PRKAR1A	Rearrangement	1	0.001	-1.55 (-15.26 - 12.16)	0.824549443	1
FGF6	Missense	2	0.001	-1.08 (-10.77 - 8.61)	0.826820271	1
NOTCH4	Truncation	6	0.004	0.62 (-4.98 - 6.23)	0.827009681	1
KMT2C	Missense	29	0.017	-0.28 (-2.85 - 2.29)	0.829659074	1
ETV6	Deletion	2	0.001	-1.06 (-10.76 - 8.64)	0.830523502	1
CDH1	Missense	3	0.002	0.86 (-7.06 - 8.78)	0.831668075	1
KDM6A	Rearrangement	1	0.001	1.48 (-12.23 - 15.2)	0.832046885	1
CBFB	Missense	1	0.001	-1.48 (-15.19 - 12.23)	0.832406984	1
MAP2K4	Missense	1	0.001	-1.47 (-15.18 - 12.24)	0.833204296	1
RAD51B	Deletion	38	0.023	-0.24 (-2.49 - 2.01)	0.833902449	1
EZH2	Rearrangement	2	0.001	1.04 (-8.66 - 10.73)	0.834111343	1

NOTCH2	Missense	13	0.008	-0.41 (-4.22 - 3.41)	0.834924689	1
DNM2	Rearrangement	3	0.002	0.84 (-7.08 - 8.76)	0.83566072	1
TP53	Truncation	93	0.056	-0.17 (-1.73 - 1.4)	0.835860601	1
CHUK	Truncation	1	0.001	-1.44 (-15.14 - 12.27)	0.836806826	1
BRD4	Rearrangement	1	0.001	1.42 (-12.29 - 15.13)	0.838926921	1
ERG	Rearrangement	1	0.001	-1.41 (-15.12 - 12.29)	0.839653864	1
LRP1B	Truncation	8	0.005	0.5 (-4.35 - 5.34)	0.840417168	1
CXCR4	Rearrangement	2	0.001	-0.99 (-10.68 - 8.71)	0.841356333	1
MAP2K1	Amplification	2	0.001	-0.97 (-10.66 - 8.73)	0.844525419	1
GRIN2A	Missense	4	0.002	-0.68 (-7.54 - 6.17)	0.844744442	1
FANCL	Rearrangement	1	0.001	1.35 (-12.35 - 15.05)	0.846629098	1
FLT4	Missense	1	0.001	1.34 (-12.37 - 15.05)	0.8479601	1
NSD2	Missense	1	0.001	1.34 (-12.37 - 15.04)	0.848237151	1
KMT2C	Rearrangement	1	0.001	-1.3 (-15 - 12.41)	0.852973674	1
KEAP1	Missense	1	0.001	1.3 (-12.41 - 15)	0.853032259	1
KDM6A	Deletion	4	0.002	-0.64 (-7.51 - 6.22)	0.854548387	1
MAP2K4	Rearrangement	6	0.004	0.52 (-5.08 - 6.13)	0.854907472	1
CDKN2B	Missense	1	0.001	-1.26 (-14.9 - 12.38)	0.856509519	1
ZRSR2	Deletion	1	0.001	1.24 (-12.47 - 14.95)	0.859400311	1
STAT6	Rearrangement	2	0.001	0.86 (-8.83 - 10.56)	0.861459526	1
FANCD2	Rearrangement	1	0.001	-1.15 (-14.86 - 12.56)	0.869436488	1
RARA	Missense	2	0.001	-0.81 (-10.5 - 8.89)	0.869964473	1
RAD51B	Missense	3	0.002	0.65 (-7.26 - 8.57)	0.871420451	1
NOTCH1	Missense	7	0.004	-0.42 (-5.62 - 4.77)	0.872773163	1
MED12	Missense	79	0.048	-0.13 (-1.71 - 1.45)	0.874384517	1
CHEK2	Truncation	1	0.001	-1.1 (-14.82 - 12.61)	0.874540585	1
CRLF2	Missense	1	0.001	1.1 (-12.61 - 14.8)	0.87523835	1
BIRC3	Rearrangement	2	0.001	0.78 (-8.92 - 10.47)	0.87538431	1
AMER1	Missense	5	0.003	0.48 (-5.66 - 6.62)	0.878060619	1

CHEK2	Missense	15	0.009	-0.27 (-3.83 - 3.28)	0.879530164	1
CDKN2B	Rearrangement	1	0.001	1.05 (-12.59 - 14.69)	0.879699653	1
ERBB2	Amplification	3	0.002	-0.61 (-8.53 - 7.31)	0.879703255	1
MYCN	Missense	1	0.001	1.06 (-12.65 - 14.76)	0.879753577	1
SRC	Amplification	3	0.002	0.61 (-7.31 - 8.53)	0.880344667	1
RAD21	Missense	2	0.001	0.74 (-8.95 - 10.44)	0.880446828	1
DTX1	Missense	1	0.001	1.05 (-12.66 - 14.75)	0.880883001	1
MPL	Missense	4	0.002	0.52 (-6.34 - 7.38)	0.881297221	1
BRCA1	Deletion	1	0.001	1.04 (-12.67 - 14.75)	0.881455065	1
CSF3R	Amplification	1	0.001	-1.03 (-14.74 - 12.67)	0.882395635	1
MTAP	Rearrangement	2	0.001	0.71 (-8.99 - 10.4)	0.886191751	1
WT1	Missense	2	0.001	-0.68 (-10.38 - 9.02)	0.891048557	1
IGF1R	Missense	1	0.001	-0.93 (-14.64 - 12.78)	0.894292515	1
MITF	Amplification	11	0.007	-0.28 (-4.42 - 3.87)	0.896093204	1
BCL11B	Rearrangement	1	0.001	-0.9 (-14.61 - 12.8)	0.897103278	1
STAG2	Missense	5	0.003	0.39 (-5.74 - 6.53)	0.899784077	1
MAP3K14	Truncation	1	0.001	-0.88 (-14.59 - 12.83)	0.899945147	1
PTPN6	Missense	1	0.001	0.87 (-12.83 - 14.58)	0.900848382	1
CUX1	Deletion	1	0.001	-0.87 (-14.58 - 12.85)	0.901440692	1
NOD1	Missense	3	0.002	-0.5 (-8.42 - 7.42)	0.901946318	1
CCT6B	Truncation	3	0.002	-0.5 (-8.42 - 7.42)	0.902301285	1
PTPRO	Rearrangement	4	0.002	0.43 (-6.44 - 7.29)	0.903208874	1
ATR	Truncation	3	0.002	-0.47 (-8.39 - 7.45)	0.906731392	1
AKT2	Amplification	9	0.005	0.27 (-4.31 - 4.85)	0.907070172	1
KDR	Amplification	15	0.009	0.21 (-3.34 - 3.76)	0.907757841	1
RAD51D	Missense	1	0.001	0.81 (-12.9 - 14.51)	0.908056575	1
FLCN	Truncation	3	0.002	0.46 (-7.45 - 8.37)	0.908462431	1
ARID1A	Missense	7	0.004	-0.3 (-5.49 - 4.89)	0.908964169	1

BRCA1	Rearrangement	2	0.001	-0.56 (-10.26 - 9.13)	0.909436209	1
PALB2	Missense	7	0.004	0.29 (-4.9 - 5.48)	0.912308253	1
BCOR	Truncation	1	0.001	-0.76 (-14.44 - 12.92)	0.913032533	1
MAP3K6	Rearrangement	2	0.001	0.53 (-9.17 - 10.23)	0.914288853	1
SDHC	Missense	1	0.001	-0.73 (-14.44 - 12.98)	0.91676404	1
EWSR1	Rearrangement	2	0.001	0.51 (-9.18 - 10.21)	0.917461585	1
ZRSR2	Missense	5	0.003	0.32 (-5.82 - 6.46)	0.917966144	1
CCT6B	Missense	16	0.01	0.18 (-3.26 - 3.62)	0.91804469	1
SGK1	Missense	1	0.001	0.72 (-12.99 - 14.42)	0.918276197	1
NCOR2	Rearrangement	4	0.002	0.35 (-6.5 - 7.21)	0.919202336	1
CUX1	Truncation	1	0.001	0.69 (-13.02 - 14.41)	0.920952528	1
FHIT	Truncation	1	0.001	0.67 (-12.91 - 14.26)	0.922492795	1
STK11	Deletion	6	0.004	0.26 (-5.34 - 5.87)	0.926361946	1
FAS	Truncation	1	0.001	-0.6 (-14.3 - 13.1)	0.931887652	1
ETS1	Truncation	1	0.001	0.6 (-13.11 - 14.3)	0.93192308	1
CTNNA1	Rearrangement	2	0.001	0.42 (-9.27 - 10.11)	0.932131676	1
APC	Missense	15	0.009	0.15 (-3.4 - 3.7)	0.932819098	1
TP63	Rearrangement	1	0.001	0.57 (-13.15 - 14.28)	0.935562576	1
PTPRO	Deletion	1	0.001	0.56 (-13.15 - 14.27)	0.93581584	1
JAK2	Missense	2	0.001	-0.39 (-10.08 - 9.3)	0.937150301	1
SMARCA4	Missense	6	0.004	0.21 (-5.4 - 5.82)	0.941053784	1
ROS1	Rearrangement	2	0.001	-0.36 (-10.05 - 9.33)	0.941937917	1
MSH2	Truncation	3	0.002	-0.28 (-8.2 - 7.64)	0.944107331	1
SH2B3	Missense	2	0.001	-0.33 (-10.03 - 9.37)	0.946764245	1
FANCG	Missense	1	0.001	-0.46 (-14.17 - 13.25)	0.948059885	1
TAF15	Rearrangement	1	0.001	0.45 (-13.26 - 14.15)	0.949009373	1
HSP90AA1	Rearrangement	1	0.001	-0.44 (-14.15 - 13.27)	0.950149422	1

JARID2	Rearrangement	1	0.001	-0.43 (-14.13 - 13.27)	0.950536152	1
NOTCH1	Rearrangement	1	0.001	-0.43 (-14.14 - 13.29)	0.951486226	1
FGFR4	Missense	1	0.001	-0.42 (-14.13 - 13.29)	0.952332608	1
SDHA	Deletion	5	0.003	0.19 (-5.95 - 6.32)	0.95272278	1
CEBPA	Missense	1	0.001	-0.41 (-14.12 - 13.29)	0.952816208	1
INPP4B	Deletion	2	0.001	0.28 (-9.41 - 9.97)	0.954388768	1
ERBB3	Missense	2	0.001	0.26 (-9.44 - 9.95)	0.958364075	1
NCOR2	Deletion	2	0.001	-0.24 (-9.93 - 9.45)	0.961172984	1
SETBP1	Missense	2	0.001	-0.24 (-9.93 - 9.46)	0.9615066	1
FHIT	Missense	3	0.002	0.18 (-7.67 - 8.03)	0.964657416	1
KDM6A	Truncation	3	0.002	-0.18 (-8.1 - 7.75)	0.96512392	1
PIK3R1	Missense	7	0.004	-0.12 (-5.28 - 5.05)	0.965169266	1
PMS2	Deletion	2	0.001	0.18 (-9.51 - 9.86)	0.971627912	1
BLM	Truncation	1	0.001	0.24 (-13.47 - 13.95)	0.972754351	1
BACH1	Truncation	1	0.001	0.24 (-13.48 - 13.96)	0.972832009	1
MAPK1	Amplification	17	0.01	0.05 (-3.29 - 3.4)	0.974286215	1
ARFRP1	Amplification	3	0.002	-0.12 (-8.04 - 7.8)	0.976912366	1
IGH	Rearrangement	3	0.002	0.11 (-7.8 - 8.03)	0.977560855	1
BRD4	Truncation	2	0.001	-0.14 (-9.84 - 9.56)	0.977586231	1
ETV6	Rearrangement	5	0.003	0.07 (-6.07 - 6.21)	0.981756771	1
SDHA	Missense	7	0.004	0.05 (-5.14 - 5.24)	0.98453604	1
FOXP1	Amplification	13	0.008	-0.03 (-3.85 - 3.78)	0.98685523	1
VHL	Missense	7	0.004	0.04 (-5.15 - 5.23)	0.98716038	1
CDK12	Missense	3	0.002	0.05 (-7.87 - 7.97)	0.989621076	1
STK11	Rearrangement	4	0.002	-0.03 (-6.89 - 6.83)	0.993538331	1
WT1	Rearrangement	2	0.001	0.02 (-9.68 - 9.72)	0.996360538	1

Supplementary Table 5: HumanBase tissue-specific network analysis direct interaction with the greatest confidence

Supplementary Table 5: HumanBase tissue-specific network analysis direct interaction with the greatest confidence		
GENE1	GENE2	WEIGHT
MYC	*FBXW7*	0.734549
NF1	*FBXW7*	0.327312
DAXX	*CDKN2A*	0.293284
NF1	*RB1*	0.218863
RB1	*CDKN2A*	0.215108
RB1	*FBXW7*	0.154473
MYC	*RB1*	0.149758
BRCA2	*NF1*	0.117482
CDKN2B	*RB1*	0.092586
CDKN2B	*CDKN2A*	0.085421
BRCA2	*RB1*	0.082818
MYC	*CDKN2A*	0.080116
NF1	*MYC*	0.075296
FBXW7	*CDKN2A*	0.072678
BRCA2	*FBXW7*	0.071873
BRCA2	*MYC*	0.062145
NF1	*CDKN2A*	0.061095
NF1	*CDKN2B*	0.057755
DAXX	*NF1*	0.050498

Supplementary Table 6: OSUCCC Patient Demographics

Supplementary Table 6: OSUCCC Patient Demographics (n=40)	
Age (median [range])	61 (43-79)
Sex	
Female	30 (75%)
Male	10 (25%)
Ethnicity	
Caucasian	38 (95%)
African American	2 (5%)
ECOG	
0	18 (45%)
1	21 (52.5%)
2	1 (2.5%)
Site	
Uterus	13 (32.5%)
Retroperitoneum	12 (30%)
Extremity	6 (15%)
Pelvis	5 (12.5%)
Other	4 (10%)
Grade	
High	27 (67.5%)
Intermediate	9 (22.5%)
Unknown	4 (10%)
